# Genome-wide association and functional interrogation identified a variant at 3p26.1 modulating ovarian cancer survival among Chinese women

**DOI:** 10.1038/s41421-021-00342-6

**Published:** 2021-12-21

**Authors:** Hongji Dai, Xinlei Chu, Qian Liang, Mengyun Wang, Lian Li, Yao Zhou, Zhanye Zheng, Wei Wang, Zhao Wang, Haixin Li, Jianhua Wang, Hong Zheng, Yanrui Zhao, Luyang Liu, Hongcheng Yao, Menghan Luo, Qiong Wang, Shan Kang, Yan Li, Ke Wang, Fengju Song, Ruoxin Zhang, Xiaohua Wu, Xi Cheng, Wei Zhang, Qingyi Wei, Mulin Jun Li, Kexin Chen

**Affiliations:** 1 Department of Epidemiology and Biostatistics, National Clinical Research Center for Cancer, Key Laboratory of Molecular Cancer Epidemiology of Tianjin, Tianjin Medical University Cancer Institute and Hospital, Tianjin Medical University, Tianjin, China; 2 Department of Pharmacology, the Province and Ministry Co-sponsored Collaborative Innovation Center for Medical Epigenetics, School of Basic Medical Sciences, Tianjin Medical University, Tianjin, China; 3 Cancer Institute, Fudan University Shanghai Cancer Center, Fudan University, Shanghai, China; 4 Department of Oncology, Shanghai Medical College, Fudan University, Shanghai, China; 5 Cancer Biobank, National Clinical Research Center for Cancer, Tianjin Medical University Cancer Institute and Hospital, Tianjin Medical University, Tianjin, China; 6 School of Biomedical Sciences, LKS Faculty of Medicine, The University of Hong Kong, Hong Kong, SAR China; 7 Department of Obstetrics and Gynaecology, Hebei Medical University, Fourth Hospital, Shijiazhuang, China; 8 Department of Molecular Biology, Hebei Medical University, Fourth Hospital, Shijiazhuang, China; 9 Department of Gynecologic Oncology, National Clinical Research Center for Cancer, Tianjin Medical University Cancer Institute and Hospital, Tianjin Medical University, Tianjin, China; 10 Center for Cancer Genomics and Precision Oncology, Wake Forest Baptist Comprehensive Cancer Center, Wake Forest Baptist Medical Center, Winston-Salem, NC USA; 11 Department of Cancer Biology, Wake Forest School of Medicine, Winston-Salem, NC USA; 12 Duke Cancer Institute, Duke University Medical Center, Durham, NC USA; 13 Department of Population Health Sciences, Duke University School of Medicine, Durham, NC USA

**Keywords:** Ovarian cancer, Cancer epidemiology

## Abstract

Ovarian cancer survival varies considerably among patients, to which germline variation may also contribute in addition to mutational signatures. To identify genetic markers modulating ovarian cancer outcome, we performed a genome-wide association study in 2130 Chinese ovarian cancer patients and found a hitherto unrecognized locus at 3p26.1 to be associated with the overall survival (*P*_combined_ = 8.90 × 10^−10^). Subsequent statistical fine-mapping, functional annotation, and eQTL mapping prioritized a likely casual SNP rs9311399 in the non-coding regulatory region. Mechanistically, rs9311399 altered its enhancer activity through an allele-specific transcription factor binding and a long-range interaction with the promoter of a lncRNA *BHLHE40-AS1*. Deletion of the rs9311399-associated enhancer resulted in expression changes in several oncogenic signaling pathway genes and a decrease in tumor growth. Thus, we have identified a novel genetic locus that is associated with ovarian cancer survival possibly through a long-range gene regulation of oncogenic pathways.

## Introduction

Ovarian cancer is the second most lethal gynecological malignancy after cervical cancer in China^1^. There is a lack of effective screening methods for early detection, and approximately 70% of patients present an advanced stage, when diagnosed, contributing to the high mortality rate^2,3^. Several clinical features and epidemiologic risk factors, including patient age, health status, lifestyle behaviors, tumor characteristics, and response to treatment, have been used to predict ovarian cancer survival^4–6^. However, these factors only partially explain the observed heterogeneity of survival among ovarian cancer patients.

Genetic components have long been associated with the etiology of common cancers^7^. In family studies^8–11^ and genome-wide association studies (GWASs)^12–14^, many rare and common germline variants have been identified to confer susceptibility to ovarian cancer. Accumulating data from genome-wide scans have also reported some germline variants that may modulate ovarian cancer prognosis^12,15–19^. However, the vast majority of these reported survival associations were not statistically robust or functionally interrogated. In addition, few studies have addressed the impact of ethnicity in ovarian cancer susceptibility and outcomes^13^. Specifically for ovarian cancer, Asian women exhibit lower incidence rates and higher survival rates than western women^20,21^. Nevertheless, among 207,252 women who died of ovarian cancer worldwide in 2020, nearly one in six deaths occurred in China^22^, underscoring the importance of exploring and mechanistically understanding the causal factors for the survival of Chinese ovarian cancer patients.

Our recent GWASs of East Asian women have identified three genetic loci (i.e., 9q22.33, 10p11.21, and 6p25.2) to be associated with risk of epithelial ovarian cancer (EOC), which are single nucleotide polymorphisms (SNPs) on or near *COL15A1*, *ANKRD30A*, and *SLC22A23*^13,23^. In the present study, we analyzed germline variants for their associations with the survival of more than 2000 Chinese ovarian cancer patients and identified a hitherto unrecognized locus at 3p26.1 to be associated with the survival. Subsequent fine-mapping and molecular experiments pinpointed a potentially causal variant rs9311399 that showed an allele-specific effect on transcription factor (TF) binding and a long-range chromosome interaction with a long non-coding RNA (lncRNA). Finally, deletion of the regulatory region containing rs9311399 through the CRISPR–Cas9-based technology led to altered cancer growth.

## Results

### A genome-wide significant locus at 3p26.1 linked to ovarian cancer survival

As illustrated in the study design (Supplementary Fig. S1 and Materials and methods), we evaluated associations between germline variants and overall survival (OS) with 588 deaths from 5697 person-years in 2 large Chinese ovarian cancer GWASs: the Shanghai Ovarian Cancer Study (SOCS I and SOCS II, discovery stage) and the Tianjin Ovarian Cancer Study (TOCS I and TOCS II, validation stage). The characteristics of these ovarian cancer cases are shown in Table 1. Among all the ovarian cancer patients, 76.9% were of serous EOC, and 75.3% had an advanced stage. The mean age (±standard deviation, SD) at diagnosis was 54.2 (±10.7) years, and age and clinical stage were strongly associated with survival (both *P* < 0.0001). The median survival time (MST) was 153 and 55.9 months for patients with an early and advanced disease, respectively.

**Table 1:** Clinical characteristics of patients in each study.

Characteristics	Discovery set		Replication set		Combined (*N* = 2130)
	SOCS-I (*N* = 337)	SOCS-II (*N* = 1009)	TOCS-I (*N* = 199)	TOCS-II (*N* = 585)	
Age at diagnosis, mean (SD)	54.5 (9.9)	54.7 (10.6)	54.7 (9.6)	52.9 (11.7)	54.2 (10.7)
*FIGO stage, N (%)*					
I	13 (3.9)	80 (7.9)	32 (16.1)	142 (24.3)	267 (12.5)
II	31 (9.2)	126 (12.5)	40 (20.1)	63 (10.8)	260 (12.2)
III	258 (76.6)	676 (67.0)	103 (51.8)	350 (59.8)	1387 (65.1)
IV	35 (10.4)	127 (12.6)	24 (12.1)	30 (5.1)	216 (10.1)
*Histology, N (%)*					
HGSOC	303 (89.9)	838 (83.1)	109 (54.8)	311 (53.2)	1561 (73.3)
LGSOC	2 (0.6)	19 (1.9)	6 (3.0)	49 (8.4)	76 (3.6)
ENOC	13 (3.9)	30 (3.0)	58 (29.1)	118 (20.2)	219 (10.3)
CCOC	7 (2.1)	56 (5.6)	5 (2.5)	15 (2.6)	83 (3.9)
MOC	8 (2.4)	29 (2.9)	10 (5.0)	38 (6.5)	85 (4.0)
Other/unknown	4 (1.2)	37 (3.7)	11 (5.5)	54 (9.2)	106 (5.0)
Mortality (%)^a^	48.1	16.7	48.2	27.5	27.6
Median survival time (months)^b^	67	95	64	77	75

*TOCS* Tianjin Ovarian Cancer Study, *SOCS* Shanghai Ovarian Cancer Study, *FIGO* International Federation of Gynecology and Obstetrics, *HGSOC* High-Grade Serous Ovarian Cancer, *LGSOC* Low-Grade Serous Ovarian Cancer, *ENOC* ENdometrioid Ovarian Cancer, *CCOC* Clear Cell Ovarian Cancer, *MOC* Mucinous Ovarian Cancer.

^a^Percentage of the total deaths as recorded by the last date of follow-up.

^b^Median survival time was estimated from the KM survival curve.

**Fig. 1:**
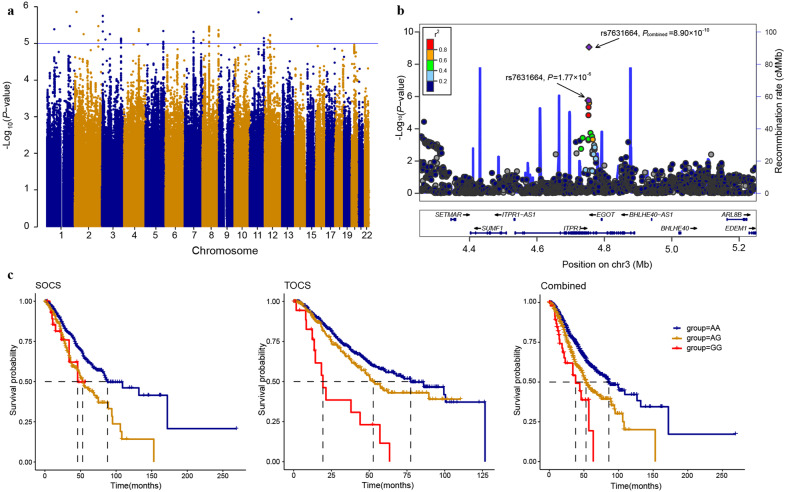
Results of genome-wide association study of survival in ovarian cancer patients. **a** Manhattan plot of results of genome-wide association analysis for survival in the discovery stage. Each point represents the negative log *P* value for an association with overall survival. The horizontal blue line shows the suggestive threshold of 1 × 10^−5^. **b** Regional plot for association statistics at the 3p26.1 region. Results are shown for SNPs in the region 500 kb up- or downstream of the leading SNP rs7631664. Each dot represents the negative log *P* value for the association statistics. The top associated SNP is colored in purple (circle in the discovery stage and diamonds in the combined stages) and the remaining SNPs are colored according to linkage disequilibrium values (*r*^2^) with the top SNP in the discovery stage. **c** Kaplan–Meier estimates of the overall survival time for ovarian cancer patients stratified by genotypes of rs7631664. The left, middle, and right represent the discovery cohort from SOCS, replication cohort from TOCS, and combined cohort, respectively. All *P* < 0.001 for the log-rank test.

In the discovery stage of 1346 ovarian cancer cases, we performed single-marker association tests across 6,577,217 SNPs by Cox proportional hazards regression analysis in an additive genetic model, with adjustment for the top three principal components of population stratification in genotyping data. The discovery GWAS yielded 78 SNPs that were associated with OS with *P* ≤ 1.0 × 10^−5^. After LD filtering (*r*^2^ < 0.2) and grouping these SNPs into intervals separated by gaps of at least 250 kb, we selected 31 independent SNPs with the lowest *P* within each interval for further replication (Fig. 1a). In the replication stage of 784 ovarian cancer patients, two independent variants, rs7631664 (*P* = 1.23 × 10^−4^) and rs142897723 (*P* = 0.054) were replicated at *P* < 0.1 with the same estimated effect direction as those in the discovery. In the joint analysis, only rs7631664 (minor allele frequency, 0.15) met the conventional genome-wide significance threshold of *P* = 5 × 10^−8^, with hazard ratios (HRs) of 1.60 (95% CI: 1.32–1.94) in the discovery stage, 1.56 (1.24–1.95) in the replication stage, and 1.58 (1.37–1.83) in the joint analysis (Fig. 1b and Supplementary Table S1). To assess the probability of the variant being a false positive, we used a Bayesian false discovery probability (BFDP) test^24^ based on a prior *P* value set to 0.0001 and an upper likely HR of 1.5. We uncovered rs7631664 and its linked variants on chromosome 3p26.1 with a cut-off value of BFDP < 0.80, as shown in Supplementary Data S1.

Kaplan–Meier plots further illustrated the association of rs7631664 genotypes with OS (Fig. 1c). The MST decreased by 33 months and 48 months for patients heterozygous and homozygous for the minor rs7631664 G allele, respectively, compared with patients homozygous for the major A allele. To assess additional independent signals at 3p26.1, we performed the analysis by conditioning on the lead SNP rs7631664 but did not observe any additional signal in this locus, when rs7631664 was adjusted for in the model (Supplementary Fig. S2).

Considering clinical features are strong factors for survival, we controlled for age and tumor stage in the Cox regression model. The association between rs7631664 and OS remained statistically significant in the adjusted model (*P*_combined_ = 1.62 × 10^−8^, Table 2). We further evaluated associations for the lead SNP rs7631664 in stratification analyses by diagnostic age, clinical stage, and histopathologic subtype. The strongest association between rs7631664 and OS was observed among younger patients (age < 55 years, HR = 1.75 and 95% CI: 1.40–2.18) and those with clear cell carcinoma (HR = 3.29, 95% CI: 1.21–8.94). However, we found no evidence for an interaction or heterogeneity after stratifying the analyses for rs7631664 by age, stage, or histology (all *P* > 0.05) (Supplementary Table S2). We also evaluated the associations of SNPs in GWAS with the survival of high-grade serous ovarian cancer (HGSOC) only. Although none of the SNPs reached genome-wide significance in the combined dataset, the SNP rs7631664 and its linked variants remained among the top hits given the reduced sample size (Supplementary Data S2).

**Table 2:** HRs and MSTs by rs7631664 genotypes in the discovery, replication, and combined GWAS samples^a^.

Genotypes	*N* (%)	MST	HR (95% CI)^b^	*P*	HR (95% CI)^c^	*P*
*Discovery*^d^			1.60 (1.32–1.94)	1.77 × 10^−^^6^	1.59 (1.31–1.93)	2.54 × 10^−^^6^
AA	962 (71.5)	88				
AG	354 (26.3)	53				
GG	29 (2.2)	46				
*Replication*			1.56 (1.24–1.95)	1.23 × 10^−4^	1.45 (1.15–1.82)	1.45 × 10^−3^
AA	540 (68.9)	78				
AG	224 (28.6)	53				
GG	20 (2.6)	19				
*Combined*			1.58 (1.36–1.83)	8.90 × 10^−10^	1.53 (1.32–1.78)	1.62 × 10^−8^
AA	1502 (70.5)	86				
AG	578 (27.1)	53				
GG	49 (2.3)	38				

*HR* hazard ratio, *MST* median survival time (months).

^a^Genotypes of rs7631664 were extracted from directly genotyping data.

^b^HR (95% CI). HR and P value was calculated using multivariable-adjusted Cox regression under a log-additive genetic model, adjusting for the top three principal components of population stratification.

^c^Cox regression model was further adjusted by age and clinical stage.

^d^One patient with missing genotype of rs7631664 in SOCS-1.

Because rs7631664 was newly identified in the present study, we performed an independent validation consisting of an additional 304 ovarian cancer patients using the TaqMan genotyping platform (Supplementary Table S3). Compared with patients with the rs7631664 A allele, patients with the G allele had a significantly higher mortality risk (adjusted HR = 1.79, 95% CI: 1.07–3.00, *P* = 0.027).

### Identification and validation of candidate causal variant

**Fig. 2:**
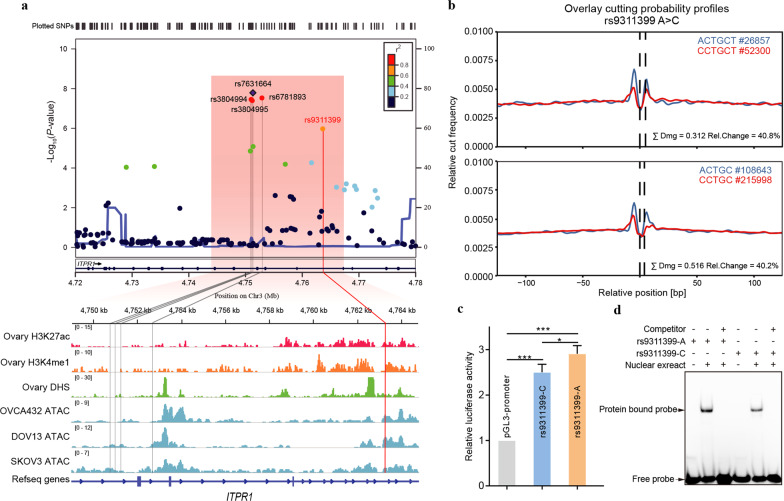
Functional annotations and prioritization of associated SNPs at 3p26.1 region. **a** Regional plot of association signals in the combined stages and annotations with ovary-specific epigenomics data, including DNase-Seq, H3K27ac, and H3K4me1 histone modification ChIP-seq profiles for primary ovary tissue (data from Roadmap), and ATAC-seq profiles from three ovarian cancer cell lines (OVCA432, DOV13, and SKOV3). The region of rs9311399 overlaps with peaks of H3K27ac, H3K4me1, and DNase I hypersensitivity sites (DHSs) measured by DNase-Seq and ATAC-seq in ovary tissues/cells. **b** TF footprint analysis using ATAC-seq. Footprint analysis showed that the rs9311399 associated sequences could affect the TF footprint. The average profiles estimated by Sasquatch software showed the highest-scoring k-mer pair (blue = reference, red = variant). The k-mers were two sequences showing the biggest difference of protein footprint with and without the investigated variant. The number represented times that k-mer within open chromatin sites (indicated by #). **c** Luciferase reporter assay using vectors containing rs9311399 in OVCA432 cells. Luciferase signals were normalized to Renilla signals (*n* = 3). Luciferase reporter assay showed the effect allele A fragment had a higher activity, compared with the non-effect allele C fragment. **d** EMSA assay showed rs9311399-A caused enhanced protein binding relative to rs9311399-C using synthetic allele-specific probes and nuclear extracts from SKOV3 cells. The rs9311399-C allele was associated with a reduction of 20% in the intensity of the shifted protein band compared to the A allele. The competitor was the unlabeled probe with the corresponding genotype. Statistical comparisons of relative luciferase activity were undertaken using Student’s *t* tests. Data are shown as means ± sd with *P < 0.05, ***P < 0.001.

We took three complement approaches to identify the likely causal variant at 3p26.1 for ovarian cancer survival. First, we applied statistical fine-mapping on all GWAS summary statistics at the linkage disequilibrium (LD) block represented by the proxy SNP rs7631664. By interrogating the ovary-specific functional annotations in the PAINTOR annotation-based fine-mapping analysis^25^, we identified a 95%-credible set (see Materials and methods) consistent of five likely causal variants, including four highly linked (*r*^2^ > 0.9) variants (i.e., rs7631664, rs6781893, rs3804994, and rs3804995) and a variant rs9311399 having *r*^2^ = 0.7 with the proxy SNP (Supplementary Table S4). All the five variants are located in the intron regions of the *ITPR1* gene, not close to the splice donor/acceptor sites, and thus, they do not impact protein-coding or splicing; rather, they may exert gene regulatory functions through altering chromatin structure and/or operating the activity of *cis*-regulatory elements. Among the five linked variants, rs9311399 coincides with the peaks of three epigenomic marks, including a DNase I hypersensitivity site and two histone modification enhancer marks (H3K27ac and H3K4me1) in normal ovarian tissue (Fig. 2a and Supplementary Table S4). The rs9311399 variant also scored the highest regulatory potential in computational prediction modeling in HaploReg^26^, GWAS4D^27^, RegulomeDB^28^, and regBase^29^ (Supplementary Data S3).

Second, to further shed light on the regulatory potential of these variants, we subsequently employed ATAC-seq, luciferase reporter assay, and EMSA to characterize these variants in ovarian cancer cell lines. We hypothesized that open chromatin in the selected ovarian cancer cell lines was representative of open chromatin in ovarian cancer because of transcriptional similarity (Supplementary Fig. S3). The ATAC-seq analyses showed that only rs9311399 resided in open chromatin regions in ovarian cancer cells (OVCA432, DOV13, and SKOV3), which was consistent with the public epigenomic profiles around the five variants in normal ovarian tissue (Fig. 2a). In the high-depth ATAC-seq profiling on rs9311399-heterozygous DOV13 cells, we used Sasquatch^30^ to perform the footprint analysis and predicted damage potential of rs9311399. By the *k*-mer scanning of cutting frequency and calculation of damaging potential between the effect and non-effect alleles, the Sasquatch identified a distinct attenuation of TF footprint for the rs9311399 A-to-C substitution, implying that the variant could affect TF occupancy in the nucleosome-free region (Fig. 2b).

We then cloned DNA sequences with the effect or non-effect allele of five corresponding credible SNPs and inserted them individually into a luciferase reporter vector with a pGL3-promoter. Upon transfection of these constructs into OVCA432 or SKOV3 cells, two variants (i.e., rs9311399 and rs7631664) showed a significant difference in the enhancer activities between effect and non-effect alleles (Fig. 2c and Supplementary Fig. S4). Cells with the effect alleles, rs9311399-A and rs7631664-G, had significantly higher luciferase expression levels, compared with those with non-effect alleles (*P* < 0.05). We next performed EMSAs to evaluate differences in protein binding between the effect and non-effect alleles. The results revealed distinct allele-specific protein binding for rs9311399 and rs6781893 (Fig. 2d and Supplementary Fig. S4).

**Fig. 3:**
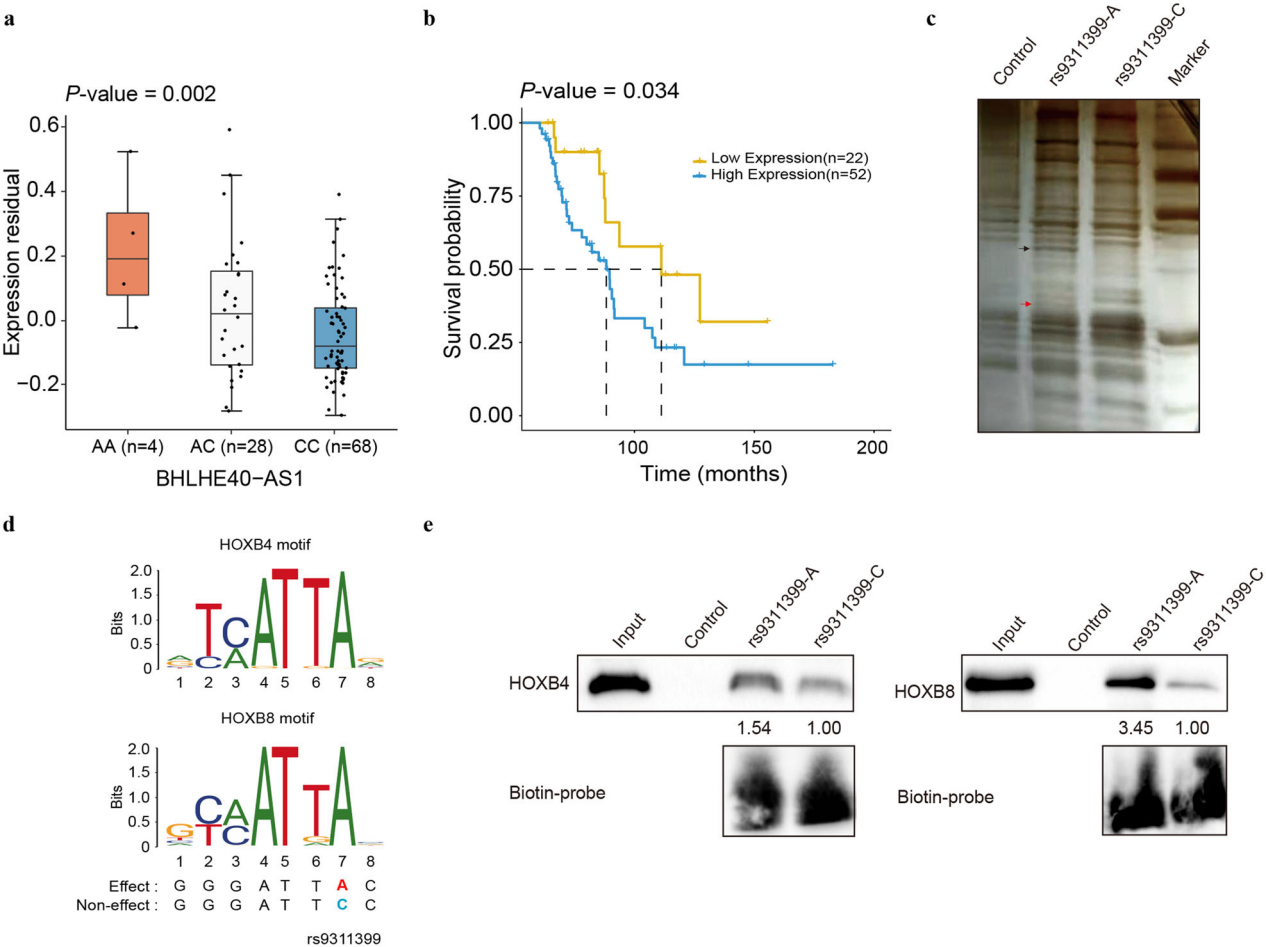
Allele-specific effect of rs9311399 on enhancer activity and protein binding. **a** eQTL analysis from Chinese ovarian cancer patients showed the rs9311399 A allele was significantly associated with high expression of *BHLHE40-AS1* in ovarian cancer tissues, *P* = 0.002 for linear regression adjusted by several confounding factors, and outlier samples in the 1.5 interquartile range (IQR) of gene expression range (measured by TPM) were removed. **b** Survival plot for patients in the TCGA dataset. The yellow line represents patients with low expression of mRNA for both *BHLHE40* and *BHLHE40-AS1* genes, while the blue line indicates those with high expression of at least one of these two genes. *P* = 0.034 for the log-rank test. **c** Silver staining of rs9311399 A/C DNA pulldown proteins and mass spectrometric analysis of rs9311399-A specific binding proteins. DNA pulldown of nuclear protein extract from SKOV3 cells with biotin-labeled rs9311399-A and rs9311399-C. The eluates were resolved on SDS-PAGE and silver-stained. The two differential bands that co-precipitated with rs9311399-A indicated by the black and red arrow were cut from the gel and followed by mass spectrometric analysis. Detailed results from the mass spectrometric analysis are shown in Supplementary Data S4. **d** Motif scanning using ATAC-seq identified HOXB4 and HOXB8 could be altered by rs9311399. **e** The rs9311399-A probe brought down more HOXB4 or HOXB8 proteins, compared with the rs9311399-C probe. DNA pulldown followed by immunoblotting with antibodies against the indicated proteins. For quantification, the intensity of HOXB4 or HOXB8 that co-precipitated with rs9311399-C was set at 1.00.

The third approach we took is the expression quantitative trait locus (eQTL) analysis. Through querying publicly available eQTL resources, such as GTEx portal^31^ and QTLbase^32^, we did not observe any significant eQTL evidence in the normal/malignant ovary tissues, except for some weak signals in other tissues (Supplementary Data S4). To ascertain the potential genetic effects of credible variants on gene expression at the phenotypically relevant ovarian tumor tissues, we performed a comprehensive eQTL mapping analysis by using data from 272 TCGA ovarian cancer samples and 112 Chinese ovarian tumor samples from the Tianjin cohort. In a linear regression model with adjustment for confounding effects from DNA copy number variants (CNVs), DNA methylation, and other clinical factors, rs9311399 was the best variant that was significantly associated with the expression level of a lncRNA *BHLHE40-AS1* in the Chinese ovarian tumor dataset (the minor A allele for increased expression, *P* = 0.002, Fig. 3a) but not in the TCGA dataset (*P* = 0.614, Supplementary Fig. S5), suggesting that rs9311399 could be a population-specific eQTL. When considering only HGSOC in the Chinese dataset, we observed significant signals for both three groups of rs9311399 genotype with *BHLHE40-AS1* (*P* = 0.005) and two groups of genotypes with *BHLHE40-AS1* (*P* = 0.009) by combining the rare homozygote and heterozygote samples (Supplementary Fig. S6). No significant eQTLs between the five credible SNPs and other neighboring genes were identified in these two datasets (Supplementary Fig. S5 and Data S5). In addition, we found that a higher expression of *BHLHE40-AS1* or *BHLHE40* (these two genes are transcribed on opposite strands) was associated with a significantly decreased OS among TCGA ovarian cancer patients with a follow-up time >60 months (*P* = 0.034, Fig. 3b). However, neither *BHLHE40-AS1* nor *BHLHE40* was independently associated with OS in all ovarian cancer patients from the TCGA dataset (Supplementary Fig. S7).

Considering the results from fine-mapping, functional annotations, primary experiment validations, and eQTL analysis, rs9311399 emerged as a top candidate causal variant for further downstream functional investigations.

### Functional investigations of the top candidate causal variant rs9311399

The above-mentioned studies showed that rs9311399 was located in an active chromatin region and may regulate the enhancer activity of its encompassing DNA sequence. To identify potential TFs that may be affected by the allele-specific effect of rs9311399, we first performed DNA pulldown of SKOV3 nuclear protein extract by using biotin-labeled rs9311399-A and rs9311399-C probes. Silver staining revealed two specific bands bound to the rs9311399-A, but not C, allele (Fig. 3c). We sequenced the proteins isolated from the two bands that were specifically co-precipitated with the biotin-labeled rs9311399-A by mass spectrometry (Supplementary Fig. S8 and Data S6). Motif scan analysis on all TFs characterized by mass spectrometry identified HOXB8 and HOXB4 as two candidate TFs that preferentially bind to the effect A allele of rs9311399 (Fig. 3d and Supplementary Table S5).

Subsequently, we performed DNA pulldown followed by immunoblotting (IB) with antibodies against the indicated proteins. The results showed that the rs9311399-A probe brought down HOXB4 (0.54 times) or HOXB8 (2.45 times) more than the rs9311399-C probe (Fig. 3e). To determine the regulation of HOXB4 or HOXB8 on *BHLHE40-AS1*, we used siRNA to silence the expression of *HOXB4* or *HOXB8* in SKOV3 cells and found that knockdown of *HOXB8* significantly decreased *BHLHE40-AS1* expression (Supplementary Fig. S9). Besides, overexpression of *HOXB8* was significantly associated with OS among TCGA ovarian cancer patients (*P* = 0.038, Supplementary Fig. S10). Thus, we propose that rs9311399 could modulate the activity of *cis*-regulatory elements by altering the binding affinities of HOXB4 or HOXB8.

**Fig. 4:**
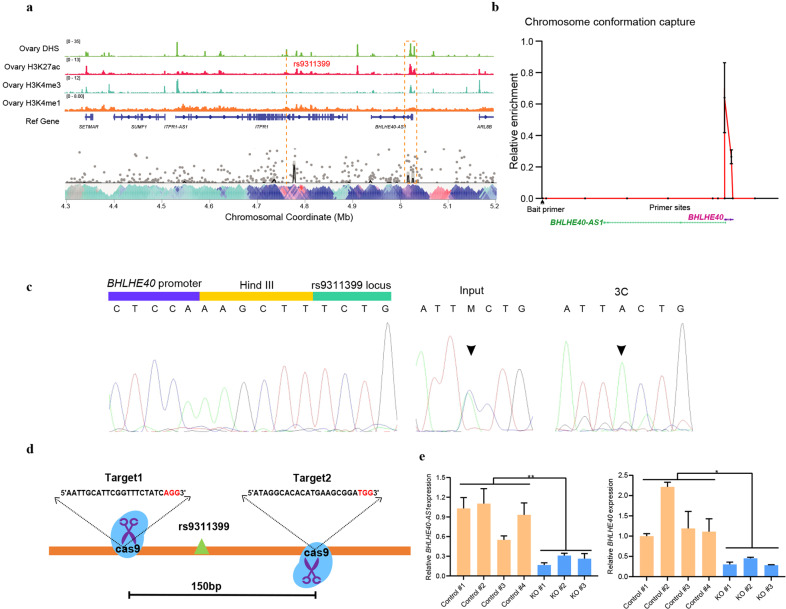
rs9311399 physically interacts with the promoter of *BHLHE40-AS1* and *BHLHE40*. **a** 4C plot and associated epigenomic annotations for rs9311399. Using the rs9311399 region as the bait in OVCA429, 4C result indicated that the rs9311399 fragment could interact with the shared promoter of *BHLHE40-AS1* and *BHLHE40*. **b** Enrichment quantification of 3C analysis confirmed chromatin interactions between *BHLHE40* promoter and rs9311399 locus. The primer sites were marked by black dots, and the bait primer was indicated by the black arrow. **c** Sanger sequencing of 3C ligation products and the rs9311399 locus from input and 3C samples. The sequence from 3C samples showed a strong allele-specific effect for rs9311399-A. The black arrow denotes the SNP location. **d** A schematic diagram shows the region surrounding the rs9311399 to be deleted by the two sgRNAs. **e** qRT-PCR analysis indicated mRNA levels of *BHLHE40* and *BHLHE40-AS1* were reduced after knockout of the rs9311399 locus among control and KO clones. Statistical comparisons of relative gene expression were undertaken using Student’s *t* tests. Data are shown as means ± sd with *P < 0.05, **P < 0.01.

Our eQTL analysis linked rs9311399 to the expression of *BHLHE40-AS1* that is located on the same chromosome albeit a 257-kb away. To investigate possible long-range regulation through chromatin looping, we first performed the circularized chromosome conformation capture (4C) using the rs9311399 region as the bait. Results from this analysis showed that the rs9311399 fragment interacted with the shared DNA region of *BHLHE40-AS1* and *BHLHE40* (Fig. 4a and Supplementary Fig. S11). Virtual 4C analysis of public data on a low-resolution ovary tissue Hi–C showed a similar result (Supplementary Fig. S12). To further validate this interaction and its likely dependence on rs9311399, we performed an additional allele-specific chromatin conformation capture assay (3C) on the rs9311399-heterozygous DOV13 cells. The 3C experiment demonstrated a distinct physical interaction between the SNP region and the shared promoter of *BHLHE40-AS1* and *BHLHE40* (Fig. 4b), consistent with the 4C results. Notably, the effect A allele of rs9311399 had a preference in 3C ligation products, suggesting that this interaction was allele-specific (Fig. 4c).

### The phenotypic impact from deletion of the rs9311399-associated enhancer

**Fig. 5:**
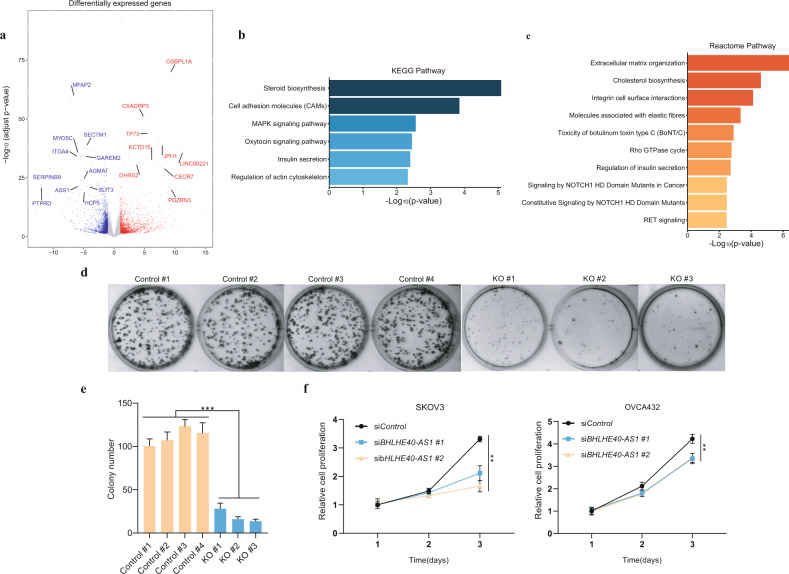
Deletion of rs9311399-associated enhancer leads to alteration in cancer-related pathways and tumorigenic capacity. **a** Volcano plot of RNA-seq for differentially expressed genes between OVCA432 WT and KO cells. **b**, **c** Pathways enrichment analysis of RNA-seq differentially expressed genes using KEGG and Reactome gene sets. **d**, **e** The representative images showed colony formation assay. Deletion of rs9311399-associated enhancer reduced colony formation in OVCA432 WT and KO cells. **f** siRNA-mediated knockdown of *BHLHE40-AS1* attenuates cell proliferation in both OVCA432 and SKOV3 cells. Statistical comparisons of colony numbers or cell proliferation were undertaken using Student’s *t* tests. Data are shown as mean ± sd with **P < 0.01, ***P < 0.001.

To investigate the molecular processes and cellular phenotypes underlying the causal variant, we used the CRISPR–Cas9 system to knock out a 150-bp intronic enhancer fragment (non-overlapping with splice elements) that only contained rs9311399 common variant in OVCA432 cells (Fig. 4d and Supplementary Fig. S13). Whole-genome sequencing on wild-type (WT) and an rs9311399 knockout (KO) clone showed no sequence difference and copy number change at both KO locus and potential off-target sites (Supplementary Fig. S14). The quantitative real-time polymerase chain reaction analysis showed that expression levels of both *BHLHE40-AS1* and *BHLHE40* were significantly decreased after the rs9311399 KO (Fig. 4e), supporting the results from 4C and 3C analyses. To further investigate downstream biological functions of the rs9311399-associated enhancer, we performed RNA-seq in WT and rs9311399 KO cells. Of the 2,940 differentially expressed genes in three KO clones versus four WT clones (adjusted *P* < 0.05 and |log2foldchange| > 1), 1,430 (48.6%) genes were upregulated, and 1510 (51.4%) were downregulated, suggesting a global effect of the rs9311399-associated enhancer on gene expression (Fig. 5a and Supplementary Fig. S15). Notably, some potential tumor suppressor genes associated with tumor growth, such as *TP73*^33^, were upregulated in the KO cells, while some likely oncogenes, such as *ITGA4*^34^, were downregulated. Additional pathway enrichment analysis showed that the differentially regulated genes were related to steroid biosynthesis, cell adhesion, and MAPK signaling pathway in the KEGG database (Fig. 5b) as well as several Notch signaling and RET signaling pathways in the Reactome database (Fig. 5c). The colony formation assay showed that tumor growth capacity was significantly decreased in the KO cells (Fig. 5d, e), while overexpression of *BHLHE40-AS1* slightly increased the colony formation ability of KO cells (Supplementary Fig. S16). Moreover, the knockdown of *BHLHE40-AS1* in both OVAC432 and SKOV3 cells also showed the reduction of cell growth (Fig. 5f). Depletion of BHLHE40-AS1 caused similar differential expression changes as in KO cells (Supplementary Fig. S17). However, the knockdown of *BHLHE40* did not significantly affect cell growth, migration, and invasion (Supplementary Fig. S18). Taken together, the removal of rs9311399-associated enhancer fragments led to an alteration in cancer-related pathways and tumorigenic capacity in ovarian cancer cells.

## Discussion

In this two-stage GWAS study of 2130 ovarian cancer patients on genetic factors for survival, we used four different bioinformatics and experimental approaches and identified a hitherto unrecognized survival-associated locus at 3p26.1, where a putative causal SNP rs9311399 is located, which was found to be significantly associated with OS of the patients. We found that, mechanistically, SNP rs9311399 may transcriptionally modulate the expression of a lncRNA *BHLHE40-AS1* through a long-range chromatin interaction. Abolition of this SNP site by chromosomal editing resulted in marked phenotypic alteration. Thus, our results posit that the 3p26.1 region is a critical site regulating RNA expression of genes to be involved in ovarian cancer progression and survival.

The region of rs9311399 overlaps with open chromatin and enhancer marks in both normal ovarian tissues and cancer cells, suggesting that this variant has a biological potential to regulate gene expression. The rs9311399 variant is located in the intron region of the *ITPR1* gene, which is surrounded by several other genes, including *SETMAR*, *EGOT*, *BHLHE40*, *BHLHE40-AS1*, and *ARL8B*. In a previous study, EGOT was confirmed to be associated with a favorable prognosis and to enhance paclitaxel sensitivity in cancer patients^35^, suggesting a link between genetic variants at 3p26.1 and cancer survival.

By eQTL, chromosome conformation capture, and CRISPR–Cas9 KO experiments, we identified a potential target gene *BHLHE40-AS1* at 3p26.1. BHLHE40-AS1 is the head-to-head antisense of a basic helix-loop-helix family member e40 (BHLHE40), while BHLHE40 (also known as DEC1 or SHARP2) belongs to the basic helix–loop–helix family that is involved in cell growth, differentiation, apoptosis, and circadian rhythm^36–39^. Several lines of evidence suggest that the lncRNA BHLHE40-AS1 is relevant in tumorigenesis. For example, BHLHE40-AS1 could drive breast cancer invasion and progression in a step-wise manner from normal, non-transformed cells to highly invasive disease^40^. Consistent with these studies, our results showed that tumor formation was significantly decreased in the rs9311399-associated enhancer KO ovarian cancer cells. Meanwhile, the knockdown of *BHLHE40-AS1* effectively suppressed tumor cell growth. However, we did not observe clear functional changes in cell growth, migration, and invasion after the knockdown of *BHLHE40*, probably due to BHLHE40 functioning as a hypoxic responsive TF that may only work under a hypoxia environment^41^. Besides *BHLHE40-AS1* and *BHLHE40*, the rs9311399-associated enhancer may also regulate other multiple target genes responsible for the observed phenotypic diversity between KO cells and knockdown of *BHLHE40-AS1*. In colony formation assay, loss of the rs9311399-associated enhancer significantly reduced tumor formation, indicating that this locus could harbor important genes for cancer growth and metastasis.

Intriguingly, our results uncovered that HOXB4 and HOXB8 are bound to rs9311399, in which an enhanced binding affinity at the effect allele was observed. Studies have found that HOXB4 is aberrantly expressed in ovarian cancer tissues and cell lines, compared with normal ovaries^42,43^, while HOXB8 is associated with shorter survival in several independent ovarian cancer follow-up studies^43,44^. These imply that the gain of HOXB4 or HOXB8 binding to rs9311399 may be a plausible driver that affects ovarian cancer progression and survival. Although our experiments showed that rs9311399 could alter its enhancer activity through allele-specific HOXB4 or HOXB8 binding and long-range interaction with target gene *BHLHE40-AS1*, the dependence of these two effects was not yet tested in the present study. In addition, we observed abnormalities of multiple signaling pathways in cancer progression, when the rs9311399-associated enhancer was knocked out. Among these oncogenic signaling pathways, the mitogen-activated protein kinase (MAPK) cascade pathway and RET signaling are critical for human cancer cell proliferation, dissemination, and resistance to drug therapy^24,45,46^. Therefore, we speculate that other cancer patients who carry the effect allele at 3p26.1 may also have a poorer prognosis as well. This hypothesis needs to be tested in other cancer patient cohorts in the future.

Because the exact etiology of ovarian cancer is currently unknown, precision prevention and treatment remain difficult to implement. There are very few studies that have reported genetic variants as predictors for ovarian cancer outcome, because many host and clinical factors may contribute to cancer patients’ survival, confounding the effect from genetic variants. On the other hand, it is yet not known to what extent the effects of genetic factors may have on survival outcomes, particularly in ovarian cancer patients. Studies have reported that *BRCA2* mutations are correlated with the survival of patients with ovarian serous carcinoma^47,48^. Previous studies also showed that germline variants at 1q22, 3q13, 9p22, 10q22, 10q23, 11q13, 11p15, and 19p13 were likely to be associated with survival outcome^12,15–19^, but none of these reached genome-wide significance. By using a hypothesis-driven pathway approach, we previously also identified a few genetic variants in the Notch signaling pathway to be associated with OS in ovarian cancer patients^49^, which was also confirmed in the present study with a much larger sample. Although limited studies examined the genetic polymorphisms with ovarian cancer susceptibility and prognosis, there is no evidence to suggest that the 3p26.1 region is associated with ovarian cancer risk. These results suggest that the genetic contribution to ovarian cancer prognosis may be independent of the contribution to disease susceptibility.

In summary, we have identified 3p26.1 locus that modulates progression in ovarian cancer cell line and thus survival of the patients. The biological mechanisms underlying the observed survival associations provide strong plausible support for the germline variant rs9311399 to be one of the causal variants, and the uncovered long-range enhancer–promoter interaction associated with this causal variant may lead to a better understanding of ovarian tumor growth and progression. Although we had adjusted for several available prognostic factors in the present study, including diagnostic age and clinical stage, it will be important to examine the utility of this genetic variant in the presence of different surgical, radiotherapy, and chemotherapy regimens in future studies with much larger patient cohorts, which may provide the scientific basis for individualized management and drug development in precision medicine of ovarian cancer once further validated.

## Materials and methods

### Study participants and design

All the participants were ethnic Chinese, who were newly diagnosed with histopathologically confirmed primary ovarian cancer. Patients with non-primary ovarian tumors were excluded. To assess the association with OS, we restricted the analysis to the patients with a definite clinical stage and a complete follow-up. The discovery stage included patients mostly from Shanghai (SOCS-I and SOCS-II), who were genotyped by various platforms. The SOCS-I study included 504 patients treated for ovarian cancer mainly at Fudan University Cancer Center (FUSCC) between 2009 and 2012^23^. The last follow-up time was December 2016. We excluded 167 patients because of either lack of clinical stage or no definite survival status up to the last follow-up. The SOCS-II study included 1047 patients treated for ovarian cancer at FUSCC between 2012 and 2015. The last follow-up time was December 2017. We excluded 38 patients because of either insufficient clinical data or insufficient follow-up data. We combined summary statistics of the SOCS-I and SOCS-II after imputation and association tests performed separately. The replication stage included patients mostly from Tianjin and Hebei (TOCS-I and TOCS-II), who were also genotyped by various platforms. The TOCS-I study included 223 patients treated for ovarian cancer at Tianjin Medical University Cancer Institute and Hospital (TMUCIH) between 2004 and 2012, and their characteristics were described previously^23^. The last follow-up date was September 2016, and we excluded 24 patients because of either insufficient clinical or follow-up data. The TOCS-II study included 417 patients diagnosed with invasive and borderline ovarian cancers at TMUCIH and additional 394 ovarian cancer patients diagnosed at the Fourth Hospital of Hebei Medical University between 2005 and 2014. The last follow-up date was January 2017. We excluded 226 patients because of either missing information on age, insufficient clinical data, insufficient follow-up data, or failure in DNA sample quality control. All the patients were followed up every 3 months after treatment completion for the first 2 years, every 6 months for the next 3 years, and annually for the following years thereafter. We combined summary statistics of the TOCS-I and TOCS-II after imputation and association test performed separately.

### Genotyping, imputation, and quality control

For TOCS-I and SOCS-I, we used a clean GWAS genotyping dataset from our previous case–control GWAS analysis^23^, in which participants were genotyped with the Illumina HumanOmniZhongHua-8 BeadChip v-6.0 (Illumina, CA, USA). Patients in SOCS-II were genotyped by Infinium Global Screening Array (GSA) BeadChip (Illumina, CA, USA). We applied extensive quality control (QC) metrics to the raw genotyping data to filter both unqualified samples and SNPs. We excluded SNPs from further analysis if they (1) did not map to autosomal chromosomes; (2) had a low call rate (< 95%) in GWAS samples; (3) had a minor allele frequency (MAF) < 0.05; and (4) had a significant deviation from Hardy-Weinberg equilibrium (*P* < 1 × 10^–5^). We also removed participants from further analysis, if they (1) had a genotyping call rate < 95% and a heterozygosity rate > 6 SD; (2) had sex discrepancies between the records and genetically inferred data; (3) were duplicate samples or probable relatives (all PI_HAT > 0.185); and (4) participants of divergent ancestry. Patients in TOCS-II were genotyped by OncoArray-500K BeadChip (Illumina, CA, USA). Details of the genotype calling for this GWAS have been described elsewhere^13^. We applied additional QC as described above for the overall variants. GWAS data QC and management were performed with PLINK^50^. No evidence of population stratification was observed in both the discovery set and replication set (lambda = 1.008 and 1.022, respectively).

Given different genotyping platforms were used, we did genome-wide imputation for each GWAS dataset and then combined all the expanded GWAS data. Specifically, we used SHAPEIT2^51^ and IMPUTE2^52^ to impute untyped SNPs by using the LD information from all samples from the 1000 Genomes Project dataset (Phase 3 data, October 2014 released). Before imputation, we excluded a small number of SNPs (≤ 3.6%) with adenine-thymine or guanine-cytosine alleles to avoid strand flipping. We performed post-imputation QC by filtering out SNPs with a missing rate > 5%, MAF < 0.05 and info score < 0.3. After filtering, approximately 6 million SNPs were used for downstream analysis.

Finally, the most significant SNP was selected and genotyped using a TaqMan genotyping platform (ABI 7900HT Real-Time PCR system, Applied Biosystems) in an additional validation dataset consisting of 304 ovarian cancer patients recruited from Zhejiang Cancer Hospital. We implemented several measures in the validation assays for QC, including (1) no template controls (NTC) were included on every assay plate, and (2) persons who performed the genotyping assays were not aware of the test or control status of the samples.

### Statistical analysis of GWAS data

The clinical stage was categorized as Stage I–IV according to the WHO 2003 classification and International Federation of Gynecology and Obstetrics (FIGO) staging system^53^. Stage subcategories coalesced for analytic purposes into summary stage categories yielding four-stage classifications (e.g., Stage IA–IC were grouped as Stage I). Clinical characteristics among patient cohorts were compared by the Student’s *t* test for continuous variables, Chi-square, or Fisher’s exact tests for dichotomous and categorical variables for bivariate analysis. OS time was calculated as the number of months from the date of diagnosis until death or the last follow-up. Follow-up was censored right on the date of death or on the date last known alive if death did not occur, whichever came first. We calculated the top three eigenvectors from directly genotyped data for each patient cohort by EIGENSOFT, which implemented the EIGENSTRAT algorithm^54^. Considering different characteristics among patient cohorts, we performed genome-wide analyses for associations with survival using Cox regression models for each of the TOCS-I & II and SOCS-I & II datasets separately. For the adjustment model, we used ProbABEL from the GenABEL suite of programs^55^, with adjustment for the top three eigenvectors in the primary Cox regression model. Given the known strong associations between age, clinical stage, and prognosis, we further adjusted for age (continuous) and FIGO stage (I–IV). Survival distributions for the most survival-associated variants were compared using the Kaplan–Meier method and log-rank test.

For the combined analysis, we used the meta-analysis summary statistics using METAL software^56^ weighted by *β* coefficients and the inverse of the corresponding standard errors (IVW). Heterogeneity of allele frequencies among the patient cohorts was assessed by *I*^2^ statistic and tested by Cochran’s *Q* test. Statistical significance was assessed at the genome-wide level (*P* = 5 × 10^−8^). For conditional analysis, we converted genotypes of the lead SNP, to the minor-allele dosage format and did Cox regression analysis with adjustment for the dosage of the SNP and top three principle components. Summary statistics were derived from a meta-analysis using the IVW method as described above.

We performed stratification analyses by age, stage, and histological type in all ovarian cancer cases. Heterogeneity was assessed by use of Cochran’s *Q*-test and *I*^2^ statistics. The test for the statistical interaction between a SNP and a prognostic factor (effect beyond additive) was performed by the inclusion of a SNP-prognostic factor cross-product term in the Cox model and assessed by use of a likelihood ratio test with 1 df.

### Annotation-based fine-mapping

We annotated SNPs in the survival-associated locus using VEP^57^ and tissue/cell type-specific epigenomes data from Roadmap Epigenomics Project (ovary primary tissue, E097)^58^, including chromatin accessibility and histone modification profiles. To determine candidate causal variants in the locus, we performed an annotation-based fine-mapping analysis using PAINTOR’s framework^25^ on all GWAS summary statistics (the adjusted model) at the SNP-located LD block. We first partitioned all variants into relatively independent LD blocks estimated by LDetect^59^. The LD information of variants in the blocks containing signals with *P*-value ≤ 5 × 10^−5^ were extracted from EAS populations of the 1000 Genomes Project^60^, while the epigenomic annotations in ovary primary tissue E097 were obtained from Roadmap^58^. We further ranked the output posterior probabilities from the largest to the smallest and determined the 95% credible set by taking the cumulative sum of descending posterior probabilities until it was at least 0.95.

### Functional variants prioritization

To prioritize the regulatory potential of fine-mapped credible SNPs in the desired locus, we first inspected functional evidence using HaploReg^26^ and GWAS4D^27^. Then, we retrieved prediction scores for credible SNPs using RegulomeDB^28^ and regBase^29^. For tissue/cell type-specific prediction, we calculated regulatory probability by cepip^61^ and GWAS4D on the matched ovary primary tissue. Finally, we prioritized the most likely functional SNPs supported by these sources of evidence.

### Ovarian tumor tissues, genotyping, RNA sequencing, and methylation profiling

Human ovarian malignant tissues were acquired from the cancer biobank of TMUCIH. We used 112 Chinese ovarian cancer patients with available tumor tissues in the tissue-based analysis. These patients included 67 serous cystadenocarcinoma, 33 endometrioid adenocarcinoma, and 12 other epithelial carcinomas. Ovarian tumor DNA of each sample was extracted and genotyped by Illumina GSA v-1 BeadChip. Total RNA of ovarian tumor tissue was extracted by the standard Trizol method. We used 2-µg RNA for RNA sequencing according to the manufacturer’s instructions. In brief, mRNA was purified from total RNA using poly-T oligo-attached magnetic beads. Sequencing libraries were generated using NEBNext^®^ UltraTM RNA Library Prep Kit for Illumina^®^ (NEB, USA) following the manufacturer’s recommendations. Sequencing was conducted with the Illumina NovaSeq 6000 platform and 150-bp paired-end reads were generated. For methylation profiling, the EZ-96 DNA Methylation-Gold kit (Zymo Research) was used for treating 500 ng of tumor DNA from each sample with sodium bisulfite. Bisulfite-treated DNA was assessed using the Illumina Infinium HumanMethylationEPIC (850 K) BeadChip and scanned by the Illumina iScan System.

Germline genotypes were processed and imputed following the same procedures as mentioned above. DNA CNVs were calculated by the Illumina cnvPartition algorithm. We filtered out CNV regions that have low confidence values (<35). Clean RNA-seq reads were obtained by removing reads containing adapter, reads containing ploy-N and low-quality reads from raw data. Index of the reference genome (hg19) was built using STAR^62^ and paired-end clean reads were aligned to the reference genome using STAR. We used RSEM^63^ to count the reads numbers mapped to each gene. Raw methylation signal intensities were processed using the bigmelon^64^ package, and the data were then normalized using the wateRmelon^65^ package.

### eQTL analysis

We performed regional eQTL analyses for all credible SNPs and expression levels of genes within the 1-MB region spanning lead SNP for the above-mentioned 112 Chinese tumor samples and 272 ovarian tumors from the Cancer Genome Atlas (TCGA). We used Transcripts Per Kilobase Million (TPM) from RSEM to quantify gene expression levels. We calculated the average of the segmented copy-number scores of gene coding regions as the gene-based somatic copy-number measures and the average methylation beta value of the probes that fall within gene coding regions as gene-based methylation measures. After removing outliers in the 1.5 interquartile range (IQR) of gene expression range (measured by TPM), we regressed for the effects of CNV, methylation, age, clinical stage, and grade. Associations between SNP genotypes and mRNA expression levels were tested by linear regression^66^ using the following model: y = SNP + CNV + meth + age + stage + grade (grade ∈ {‘low’, ‘medium’, ‘high’ }, stage∈ {‘HGSOC’, ‘ENOC’, ‘clearcell’, ‘mix’, ‘MOC’, ‘LGSOC’}).

### Cell lines and cell culture

The human SKOV3 cell line was obtained from the American Type Culture Collection (ATCC). The human OVCA429, OVCA432, and DOV13 cell lines were gifts from Dr. Wei Zhang of The University of Texas MD Anderson Cancer Center in Houston, Texas, and preserved in our lab. SKOV3 and DOV13 were cultured in a complete RPMI 1640 medium (RPMI 1640 with 10% FBS). OVCA429 and OVCA432 were cultured in a complete DMEM medium (DMEM with 10% FBS). All cells were incubated at 37 °C with 5% CO_2_.

### ATAC-seq analysis

The ATAC-Seq library was built with TruePrep DNA Library Prep Kit V2 (Vazyme) as previously described^67^. For ATAC-seq, 5 × 10^4^ cells (OVCA432, DOV13, and SKOV3) were harvested and washed by PBS. Cells were re-suspended in the lysis buffer (10 mM Tris-HCl pH 7.5, 10 mM NaCl_2_, 3 mM MgCl_2_, 0.05% NP40). The lysates were centrifuged for 3 min at 1500 G, 4 °C. The supernatants were carefully removed. Transposition reaction mix, which consisted of 10 μl of 5 × TTBL, 5 μl of TTE Mix V50 and 35 μl of ddH_2_O, was used to re-suspend nuclei pellet and incubated at 37 °C for 30 min. The transposed DNA was purified by VAHTS DNA Clean Beads (Vazyme) and PCR-amplified with the following mixture: 24 μl of purified DNA, 10 μl of 5 × TAB, 5 μl of PPM, 5 μl of N5 primer, 5 μl of N7 primer, and 1 μl of TAE. The thermal cycle was as follows: 72 °C for 3 min; 98 °C for 30 s; and thermocycling at 98 °C for 15 s, 60 °C for 30 s and 72 °C for 3 min; following by 72 °C 5 min. The amplified ATAC-Seq library was purified with VAHTS DNA Clean Beads and eluted with 30 μl ddH_2_O.

Sequencing was conducted using the Illumina NovaSeq 6000 platform and 150 bp paired-end reads were generated. Clean reads were obtained by removing reads containing adapter, reads containing ploy-N and low-quality reads from raw data. We used MACS^68^ to generate profile signals and call peaks.

### Motif scanning and TF footprint analysis

For potential SNP-associated TFs appeared in mass spectrometry assay, we collected their motifs from JASPAR^69^ and CIS-BP^70^ database. We took 30 bp of the surrounding sequence and constructed the mutated sequences for alternative alleles. We scanned the paired sequences using PWMSCAN^71^ and measured the score of binding affinity change using the log-odds of probabilities. For TF footprint analysis, we generated high-depth ATAC-seq for DOV13 (rs9311399 heterozygote cell line) and used Sasquatch^30^ to estimate and visualize the effects of rs9311399 on TF binding.

### Luciferase reporter assay

DNA fragments containing SNPs of the candidate causal variants were amplified from OVCA432 DNA. The amplified DNA fragments were inserted into the SacI and Nhe I sites of the pGL3-promoter vector. Different alleles were introduced into the vector by the PCR-based mutagenesis method. OVCA432 or SKOV3 cells were transiently co-transfected with the pGL3-promoter containing SNP with pRL-CMV Renilla luciferase reporter as a reference. The cells were harvested 24–48 h after transfection, and luciferase activities were measured by a Dual-Glo Assay System (Promega).

### Electrophoretic mobility shift assay (EMSA)

EMSA assays were performed using the LightShift Chemiluminescent EMSA Kit (Thermo Fisher Scientific). Nuclear proteins from SKOV3 cells were extracted using NE-PER Nuclear and Cytoplasmic Extraction Reagents (Thermo Fisher Scientific). 5′-biotinylated double-stranded DNA oligonucleotides corresponding to different alleles of SNPs were generated by denaturing equal amounts of complementary oligonucleotides for 15 min at 95 °C, followed by cooling to room temperature. The probe was incubated with 4 μg of nuclear proteins in 10 mM Tris pH 7.5, 55 mM KCl, 1 mM DTT, 5% glycerol, 0.05% NP40, 2.5 mM MgCl_2_, 0.25 mM EDTA, 1 µg of poly (dI–dC), at 4 °C for 1 h. For competition assays, a 200-fold amount of unlabeled probe was added in the binding mixture reactions. Reactions were then resolved on a 6% non-denatured polyacrylamide gel electrophoresis at 100 V for 90 min, followed by transferring to a nylon membrane. The transferred DNA was cross-linked to the membrane for 15 min under 254 nm UV-light. Biotin-labeled probes were detected using a luminol/enhancer solution and a stable peroxide solution according to the manufacturer’s protocol.

### DNA pulldown assay

5′-biotinylated double-stranded DNA oligonucleotides were mixed with SKOV3 nuclear extract in binding buffer containing 10 mM Tris pH 7.5, 55 mM KCl, 1 mM DTT, 5% glycerol, 0.05% NP-40, 2.5 mM MgCl_2_, 0.25 mM EDTA. Reactions were incubated at 4 °C for 6 h, followed by adding the streptavidin sepharose beads (GE) into the mixture for 2 h to bind the oligonucleotides–protein complex. The DNA-coupled beads were washed three times with the binding buffer. Beads were resuspended in a 20 μl sodium dodecyl sulfate (SDS) sample buffer. Finally, proteins were separated on a 12% SDS polyacrylamide gel followed by silver staining or IB analysis with the indicated antibodies.

### Mass spectrometry assay

The interested bands were excised from the gel, followed by in-gel tryptic digestion. The resulting peptides were loaded onto a homemade reversed-phase analytical column on an EASY-nLC 1000 UPLC system followed by tandem mass spectrometry (MS/MS) in Q Exactive^TM^ Plus (Thermo). The electrospray voltage 2.0 kV was applied. The m/z scan range was between 350 and 1800 for a full scan, and intact peptides were detected in the Orbitrap at a resolution of 70,000. All MS/MS data were processed using Proteome Discoverer 1.3. Trypsin was specified as a cleavage enzyme allowing up to 2 missing cleavages. A mass error was set to 10 ppm for precursor ions and 0.02 Da for fragment ions. Carbamidomethyl on Cys was specified as fixed modification and oxidation on Met was specified as variable modification. Peptide confidence was set at high, and peptide ion score was set >20.

### Circularized chromosome conformation capture (4C) assay

The 4C experiment was performed by setting rs9311399 as bait^72^. We used *Hin*dIII as the first restriction enzyme and *Nla*III as the second restriction enzyme. Briefly, OVCA429 cells were counted carefully, and 1 × 10^7^ cells were used for the 4C experiments. After cross-linking with 2% formaldehyde, cells were lysed with cold lysis buffer (50 mM Tris-HCl pH 7.5, 150 mM NaCl, 5 mM EDTA, 0.5% NP-40, 1% Triton X-100 and 1× complete protease inhibitors [Roche]), digested with *Hind*III and ligated with T4 DNA ligase. Then, the samples were second digested with *Nla*III and ligated with T4 DNA ligase. The ligated samples were purified using the QIAquick PCR Purification Kit (QIAGEN). The 4C-seq fragments were generated by PCR using a high-fidelity DNA polymerase (Vazyme), and then the DNA library was constructed by Illumina library preparation Kit (Vazyme, ND-607). The 4C-seq libraries were sequenced on the HiSeq X Ten Platform. We used 4C seqpipe^73^ to analyze and visualize the interaction spectrum. We also used the virtual 4C function in the 3D Genome Browser to confirm identified long-rang interaction^74^.

### Chromatin conformation capture (3C) assay

The 3C experiment was performed according to desired DNA fragments in 4C results^75^. DOV13 cells (1 × 10^6^) were cross-linked with formaldehyde and lysed by 50 mM Tris pH 7.5, 150 mM NaCl, 5 mM EDTA, 0.5% NP-40, 1% Triton X-100. Nuclei were digested with 200U HindIII (NEB) at 37 °C overnight. The DNA was ligated using T4 ligase (NEB) overnight at 37 °C. Subsequently, DNA was purified by phenol-chloroform. Control template generated by a mixture of equal amounts of different PCR products was digested with HindIII and followed by ligation and purification. The PCR products were confirmed by Sanger sequencing. Allele specificity for 3C was determined by the signal at rs9311399. We quantified 3C and control PCR products and estimated cross-linking frequencies between the anchor and test fragments.

### Generation of rs9311399-associated enhancer KO cells

The rs9311399 KO cells were generated from OVCA432 cells with the CRISPR-Cas9 System (Cas9-2hitKO) according to the manufacturer’s instructions (HedgehogBio, Shanghai, China). Target guide RNAs (target 1, 5′-CACCGAATTGCATTCGGTTTCTATC-3′; target2, 5′-CACCGATAGGCACACATGAAGCGGA-3′) were predicted by CRISPOR^76^. OVC432 cells (6 × 10^5^) were transfected with 2 μg of a CRISPR–Cas9 vector carrying two guide RNA-expressing cassettes and empty vector, respectively, in 6-well tissue culture plates for 2 days. The cells were transferred to 10-cm dishes and cultured with a medium containing puromycin (1 μg/ml) for a week. The culture medium was changed every 2–3 days. Individual cell colonies were isolated by limiting dilution. After 2 weeks, the cells were observed under a microscope. Cells from those wells containing only one cell colony were selected and allowed to expand from a 96-well plate to a 12-well plate. Genomic DNA of the cells was extracted. KO efficiency was assessed by PCR and verified by genomic DNA sequencing. To determine the genotypes of KO cells, the rs9311399 region was amplified from OVCA432 KO DNA, and DNA fragment was inserted into a pMD20-T vector (Takara) by TA cloning. After transformation into DH-5α competent cells, DH-5α colonies were selected to determine the genotypes by Sanger sequencing. Independent clones with both wild-type genotype (control) and rs9311399-associated enhancer homozygous deletion were used to perform RNA-seq and phenotype experiments.

### CRISPR off-target analysis

We used GATK v4.1.3^77^ for SNV/Indels calling and CNVkit^78^ for copy number variation calling. We compared SNV/Indels in ±500 bp around rs9311399 and ± 20 bp around potential off-target sites predicted by CRISPOR.

### Differential expression and pathway analysis

RNA-seq was performed as described above on four wild-type clones and three rs9311399 KO OVCA432 clones, and each clone was sequenced with two technical replicates. Differential expression analysis of RNA-seq data was performed using the DESeq2^79^. Genes with an adjusted *P* < 0.05 and |log2foldchange| > 1 found by DESeq2 were assigned as differentially expressed genes. Gene-set enrichment analyses for KEGG^80^ and Reactome^81^ pathways were performed by WebGestalt^82^.

### Colony formation assay

OVCA432 control cells and OVCA432 KO cells were detached with 0.25% Trypsin–EDTA, centrifuged, and re-suspended in DMEM with 10% FBS and counted. About 1 × 10^3^ cells (OVCA432 and OVCA432 KO) were seeded into 6-well plates in triplicate. Cells were cultured for 10 days at 37 °C in 5% CO_2_. Colonies were washed with PBS three times and fixed with cold methyl alcohol for 10 min. Colonies were stained with 0.01% crystal violet for 10 min. The number of colonies containing more than 50 cells was counted. All the experiments were repeated twice.

### Lentivirus transduction

The lentiviruses, produced by GenePharma, were used to transduce OVCA432 KO cells. Stable cells expressing *BHLHE40-AS1* and control cells were selected by puromycin for at least 1 week.

### siRNA knockdown

All siRNAs were synthesized by GenePharma. siRNAs were transfected by Lipofectamine RNAiMAX (Thermo Fisher Scientific). The final concentration of the siRNA was 50 nM, and cells were harvested 24 h after transfection for the proliferation assay.

### Proliferation assay

Transfected OVCA432 and SKOV3 cells were plated in 12-well plates in triplicate. At the indicated time, cells were washed with PBS to remove the dead cells. Cells of each well were trypsinized, and cell number was determined by cell counting using a hemocytometer. Relative cell growth was normalized to the cell number of Day 1.

## Supplementary information

Supplementary information

Supplementary Data S1

Supplementary Data S2

Supplementary Data S3

Supplementary Data S4

Supplementary Data S5

Supplementary Data S6

## Data Availability

All sequencing and microarray data that support the findings of this study have been deposited in the Chinese National Genomics Data Center Genome Sequence Archive (GSA) and are accessible through the GSA Series accession number CRA002378. The other relevant data that support the findings of this study are available from the corresponding author upon request.
