# Supplementary material for: Genome-wide association and functional interrogation identified a variant at 3p26.1 modulating ovarian cancer survival among Chinese women
Source: Cell Discov. 2021 Dec 21;7:121. doi: 10.1038/s41421-021-00342-6 (PMC8688503; doi:10.1038/s41421-021-00342-6)
Supplement: Supplementary file 1 — Supplementary information [file 41421_2021_342_MOESM1_ESM.pdf]

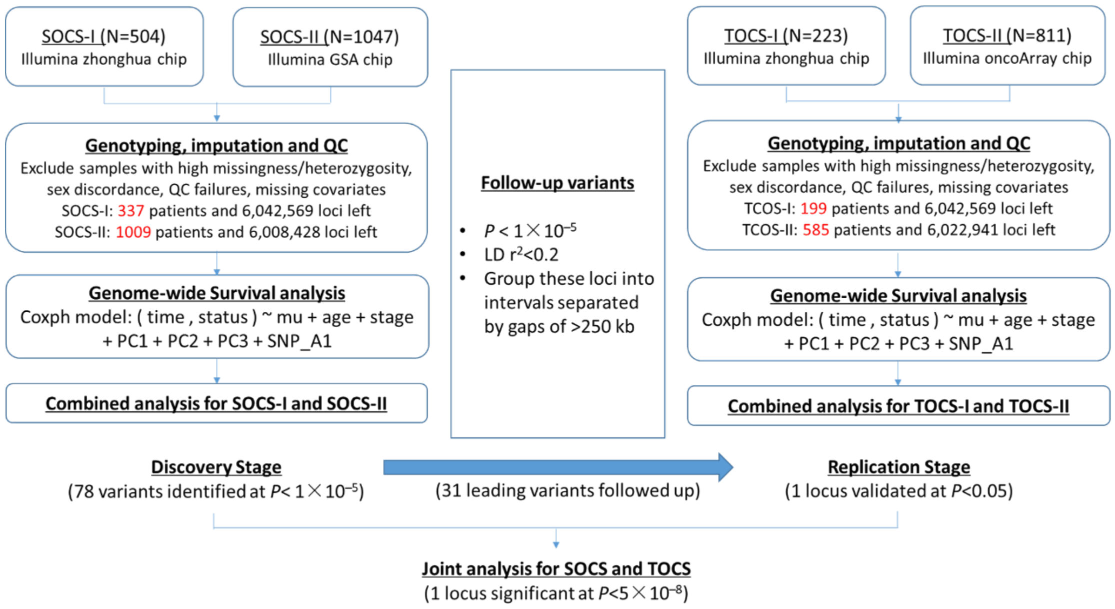

Supplementary Fig. S1. Study design. Abbreviation: SOCS, Shanghai Ovarian Cancer Study; TOCS, Tianjin Ovarian Cancer Study; PC, principal components of population stratification; LD, linkage disequilibrium.

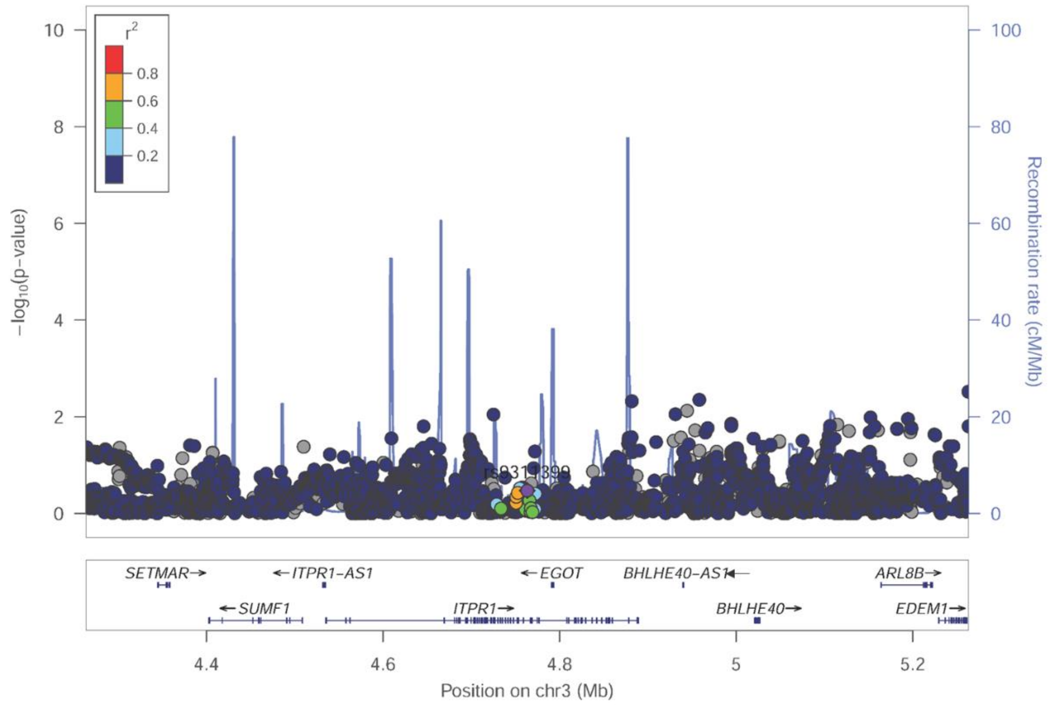

Supplementary Fig. S2. LocusZoom plot of 3p26.1 locus, conditional on the leading SNP rs7631664. No SNPs in the 3p26.1 locus (within 1M region) showed genome-wide significant association in the conditional analysis.

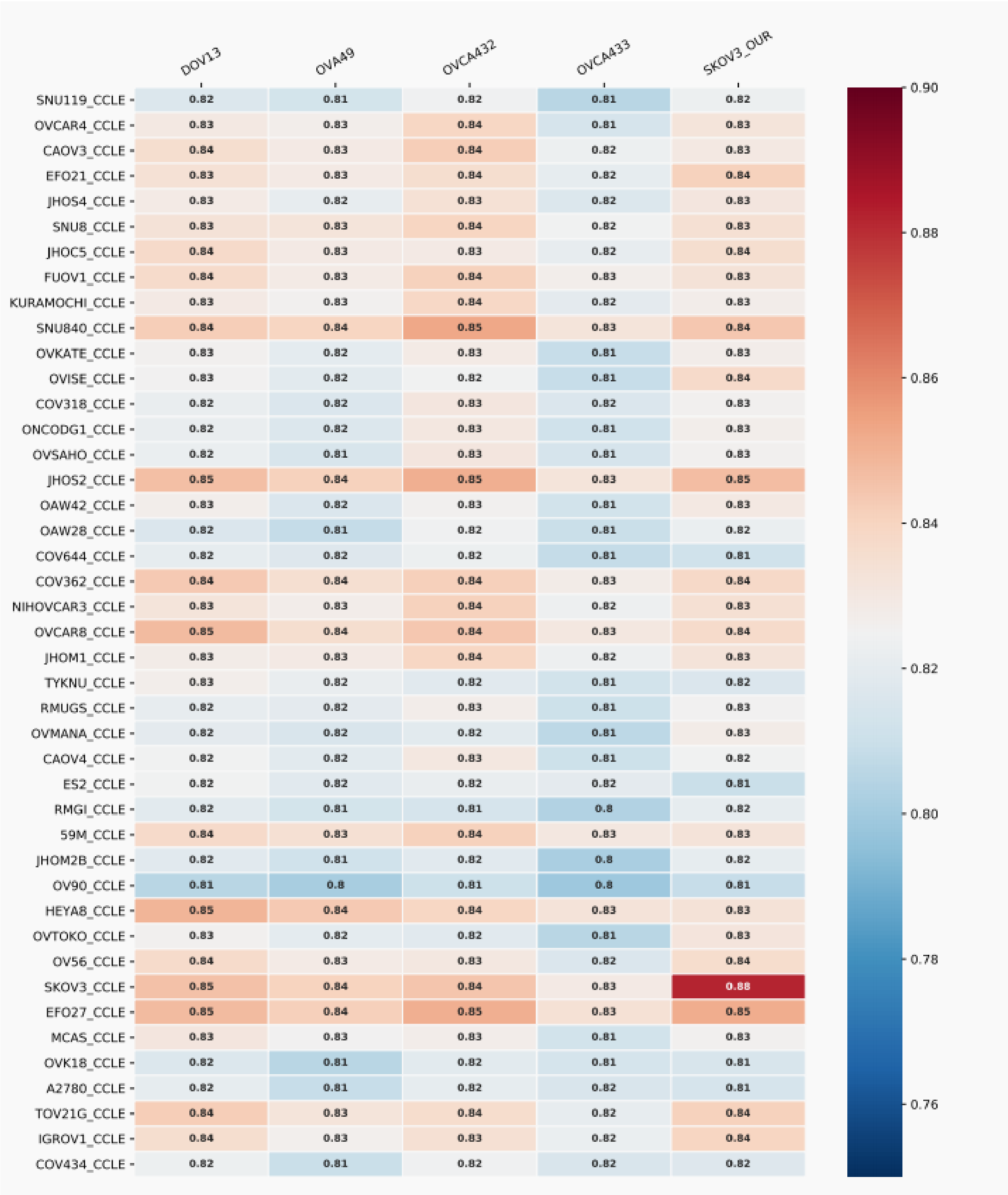

Supplementary Fig. S3. Heat map of Spearman's correlation analysis among the studied cell lines and CCLE cell lines. To evaluate the suitability of ovarian cancer cell lines used in the current study, we compared gene expression profiles between five cell lines commonly used in our lab (OVCA432, OVA433, OVA49, DOV13 and SKOV3) and 43 ovarian cancer cell lines (Domcke, S., Sinha, R., Levine, DA et al . Nat. Commun. 4, 2126 (2013)) from CCLE. The result from Spearman's correlation analysis showed that the expression patterns of our cell lines used in this study were similar to most of CCLE ovarian model cell lines (Spearman's correlation coefficients range from 0.79 to 0.89).

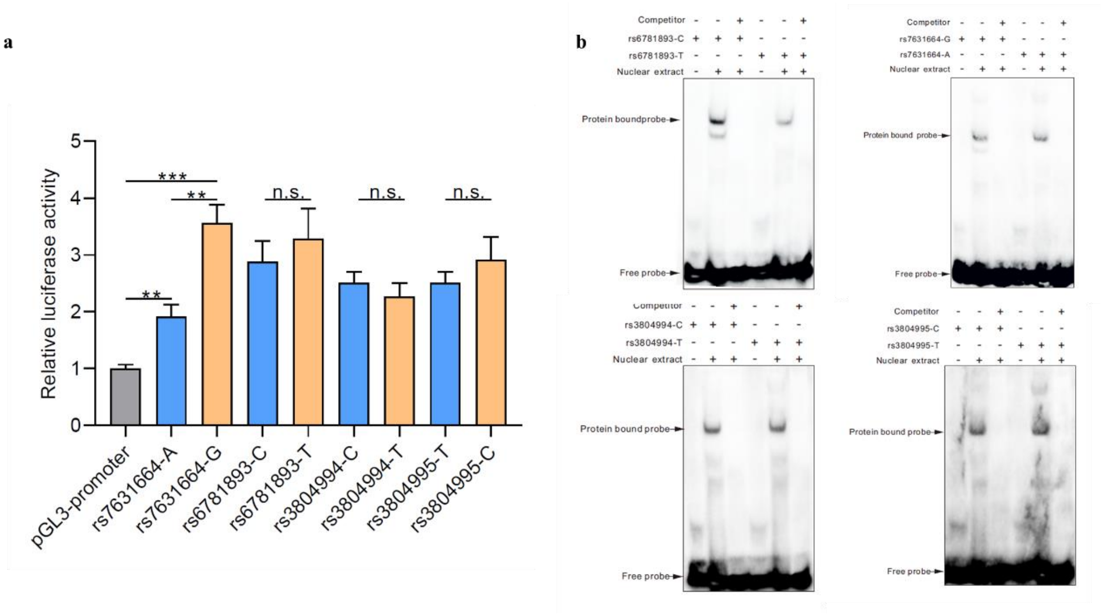

Supplementary Fig. S4. Luciferase assay and EMSA results for other linked variants. a Luciferase reporter assays using vectors containing rs6781893, rs7631644, rs3804995 or rs3804994 were performed in SKOV3 cells. Luciferase signals were normalized to Renilla signals (n = 3). Luciferase reporter assay showed the effect allele-G fragment of rs7631644 had higher activity compared to the non-effect allele-A fragment. No significant result was observed for other three variants. **b** EMSA assay showed effect allele probe of rs6781893 caused increased protein binding relative to non-effect allele probe. There were no significant changes in protein bindings for other three variants. Statistical comparisons of relative luciferase activities were undertaken using Student's *t*-tests. Data are shown as mean±s.d with $^{**}P < 0.01$, $^{***}P < 0.001$, n.s., not significant ($P > 0.05$).

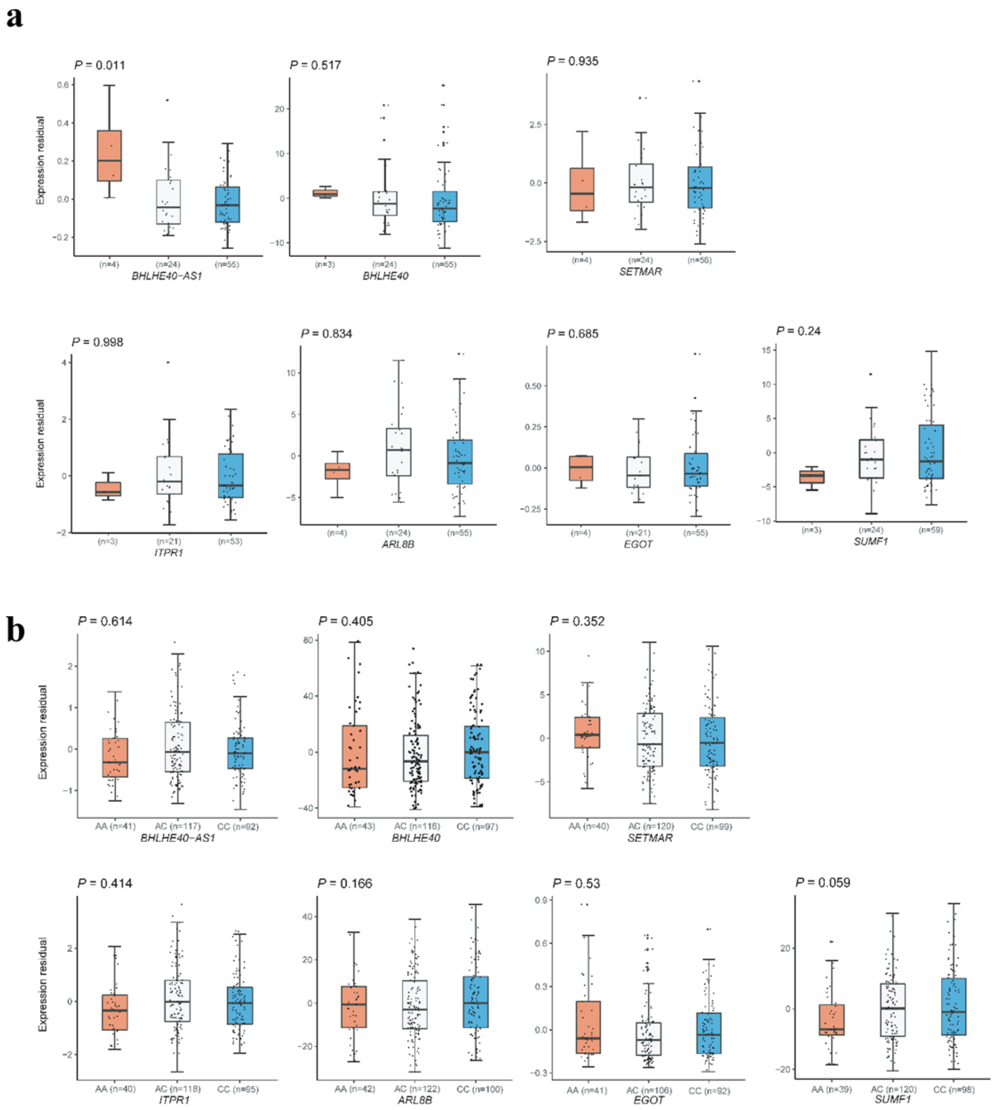

Supplementary Fig. S5. eQTL analysis in the Chinese dataset and TCGA dataset. Association between genotype of rs9311399 and neighboring gene expression was tested by linear regression and corrected for somatic copy number variation and methylation. **a** the Chinese dataset. **b** the TCGA dataset.

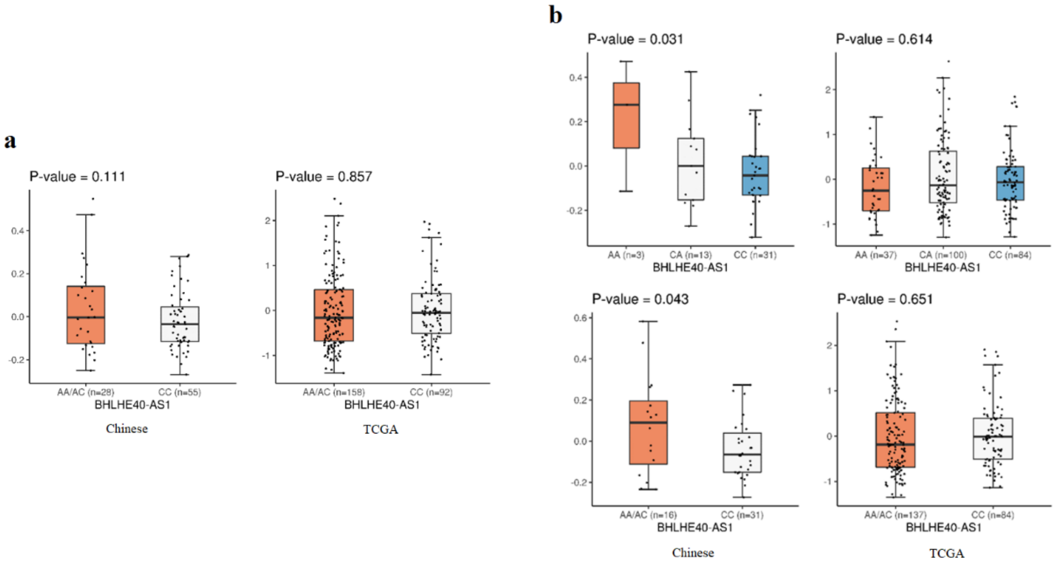

Supplementary Fig. S6. eQTL analysis for rs9311399 using different group strategies. The eQTL analysis on BHLHE40-AS1 by **a.** combining the rare homozygote and heterozygote samples in both Chinese and TCGA datasets. **b.** only high-grade serous in both Chinese and TCGA datasets.

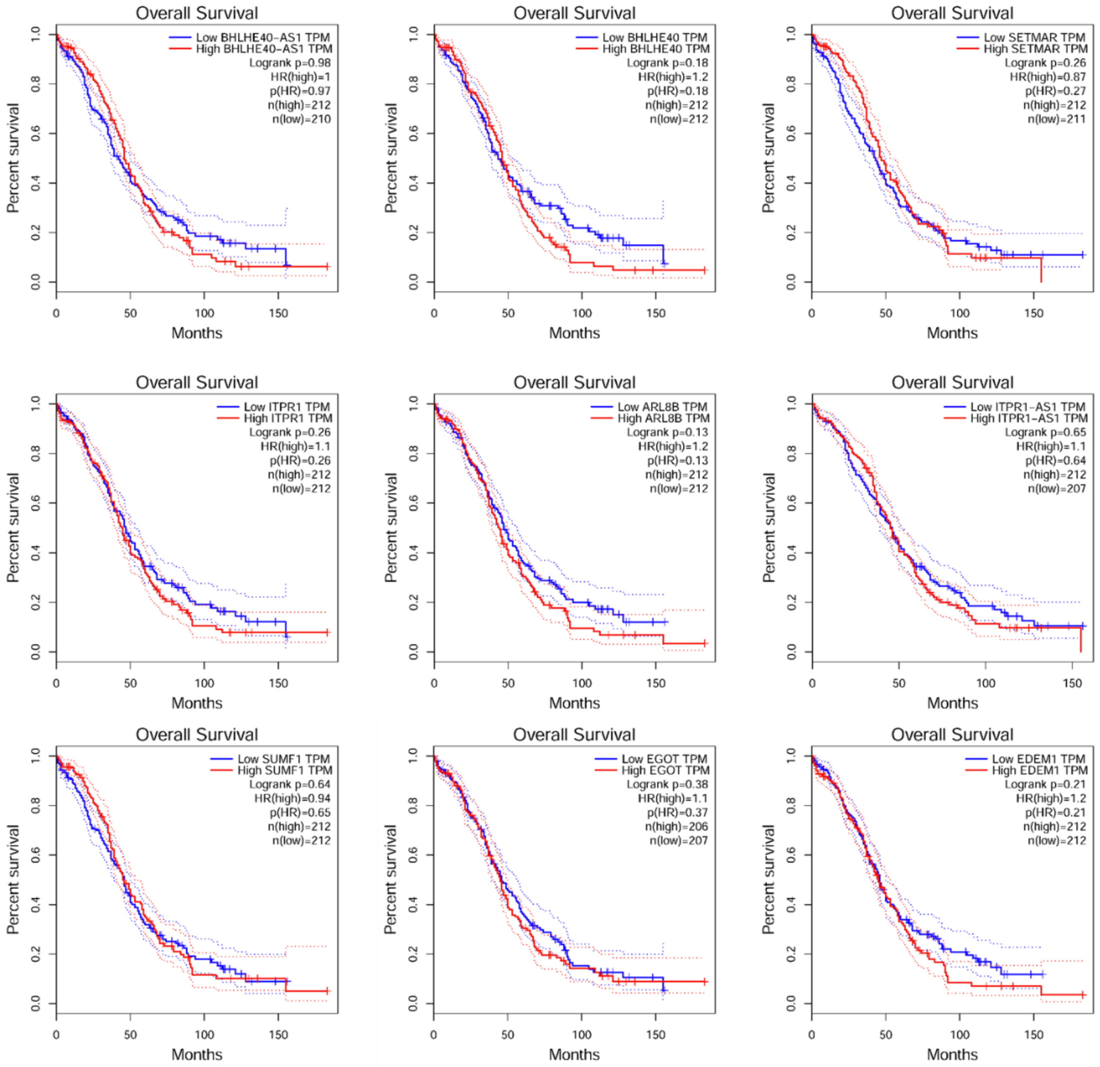

Supplementary Fig. S7. Survival analysis for risk locus surrounding genes at 3p26.1 in the TCGA dataset. Overall survival (OS) analyses were performed by GEPIA using log-rank test among TCGA ovarian cancer patients. No significant association was found between selected gene expression and overall survival time (*P* > 0.05).

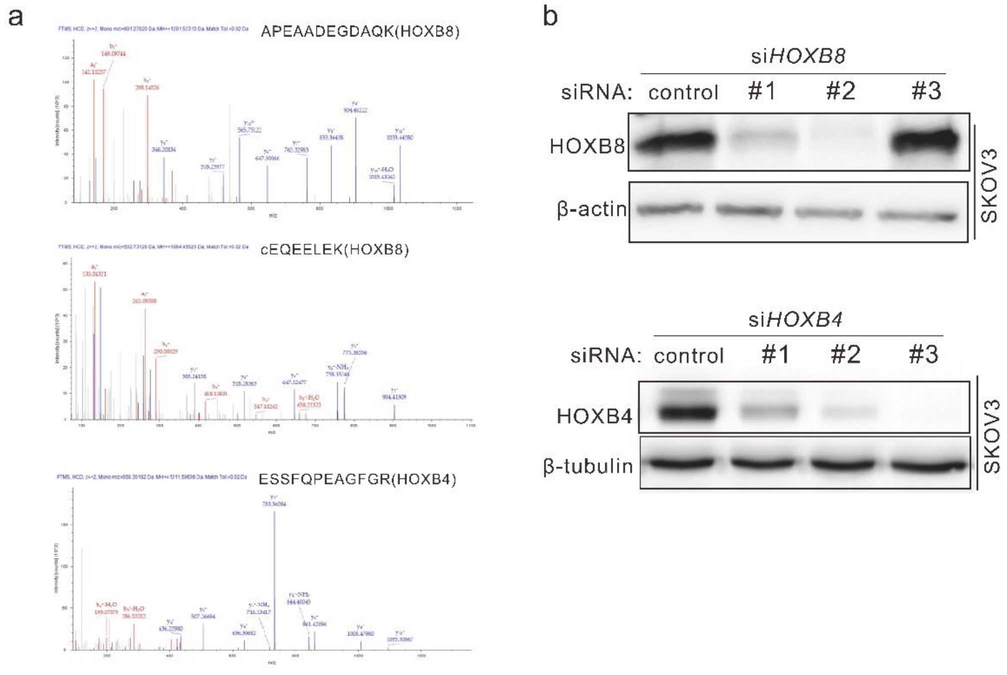

Supplementary Fig. S8. The mass spectrum and antibody specificity of HOXB8 and HOXB4. a The mass spectrum of HOXB8 and HOXB4 unique peptides. **b** The specificity of HOXB8 and HOXB4 antibodies tested by knockdown experiments.

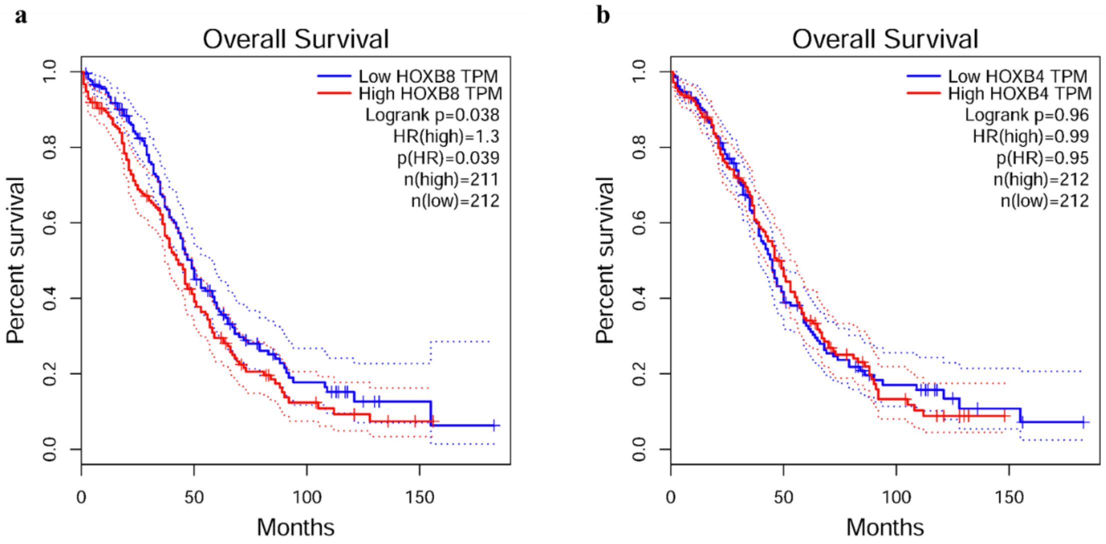

Supplementary Fig. S9. Survival analysis for HOXB8 and HOXB4 in the TCGA dataset. Overall survival (OS) analyses were performed by GEPIA using log-rank test among TCGA ovarian cancer patients. **a** High expression of HOXB8 significantly decreased overall survival time. **b** No significant association was found between HOXB4 expression and overall survival time.

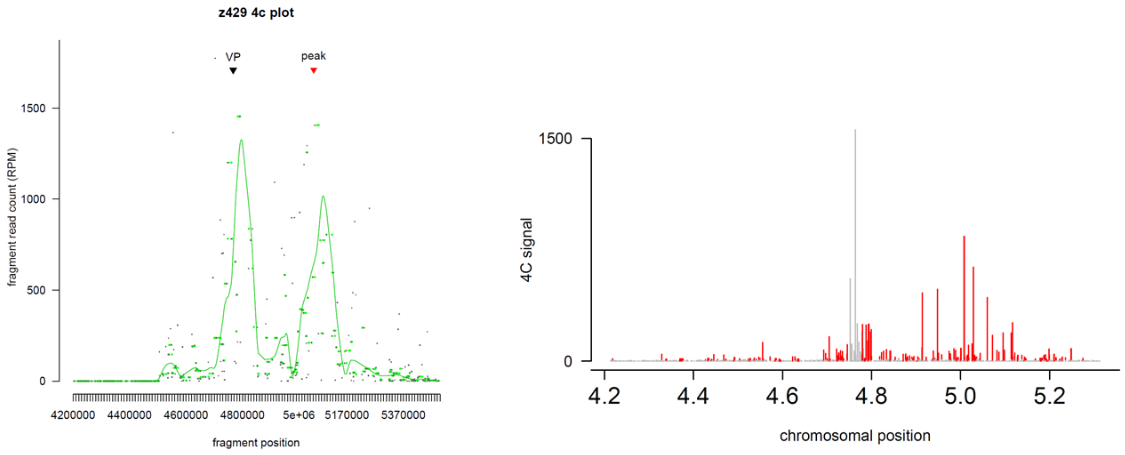

Supplementary Fig. S10. The interaction signal between rs9311399 and the promoter of BHLHE40-AS1/BHLHE40 estimated by basic4C and peakC. The highest signal from both basic4C and peakC is located in the promoter of BHLHE40-AS1/BHLHE40.

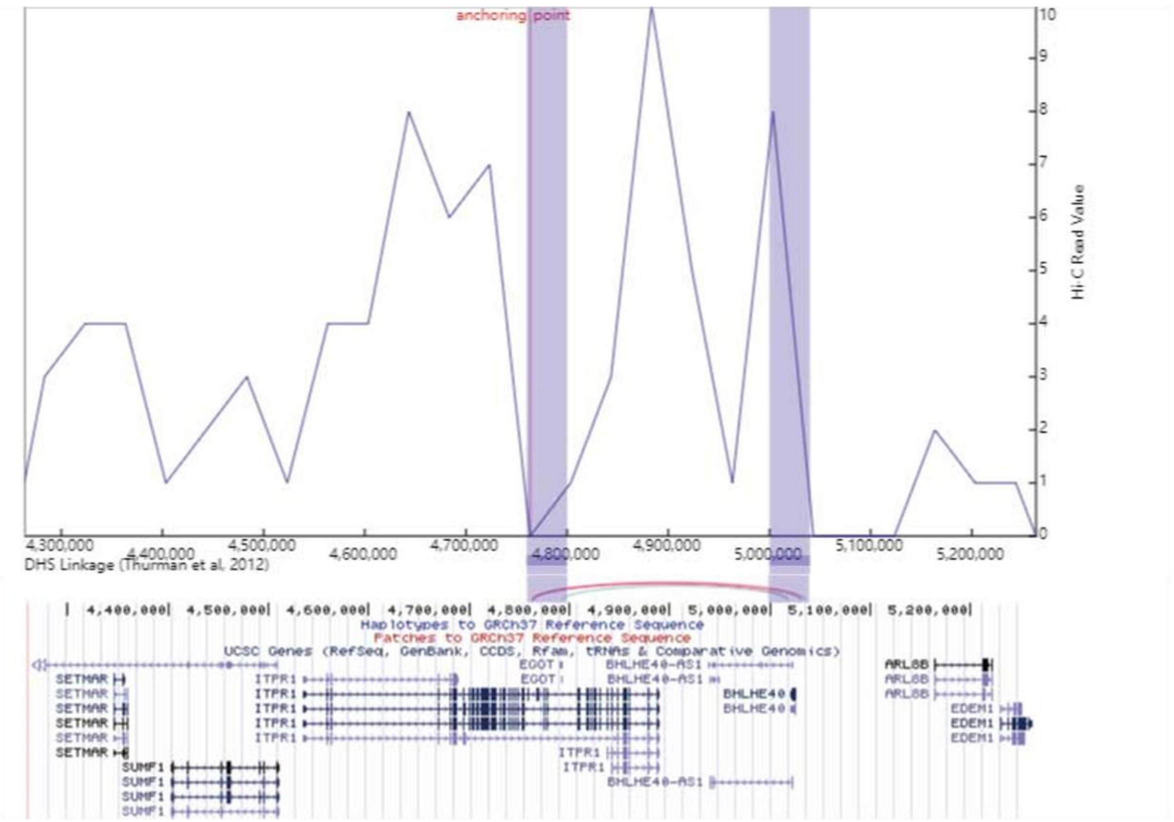

Supplementary Fig. S11. Virtual 4C analysis using public ovary Hi-C data. Ovary virtual 4C analysis in the 3D Genome browser showed that rs9311399 (viewpoint) could interact with *BHLHE40* promoter. Ovary tissue Hi-C with 40kb resolution was obtained from GSM2322546.

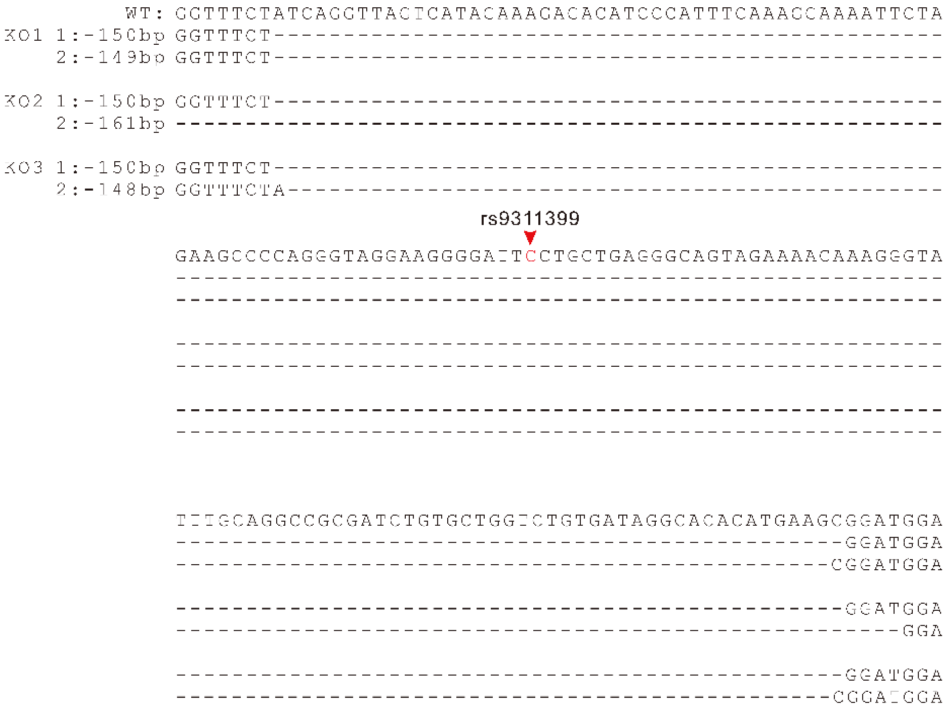

Supplementary Fig. S12. Sanger sequencing results of OVCA432 KO cells by CRISPR-Cas9 system. Deletions were indicated by dashes.

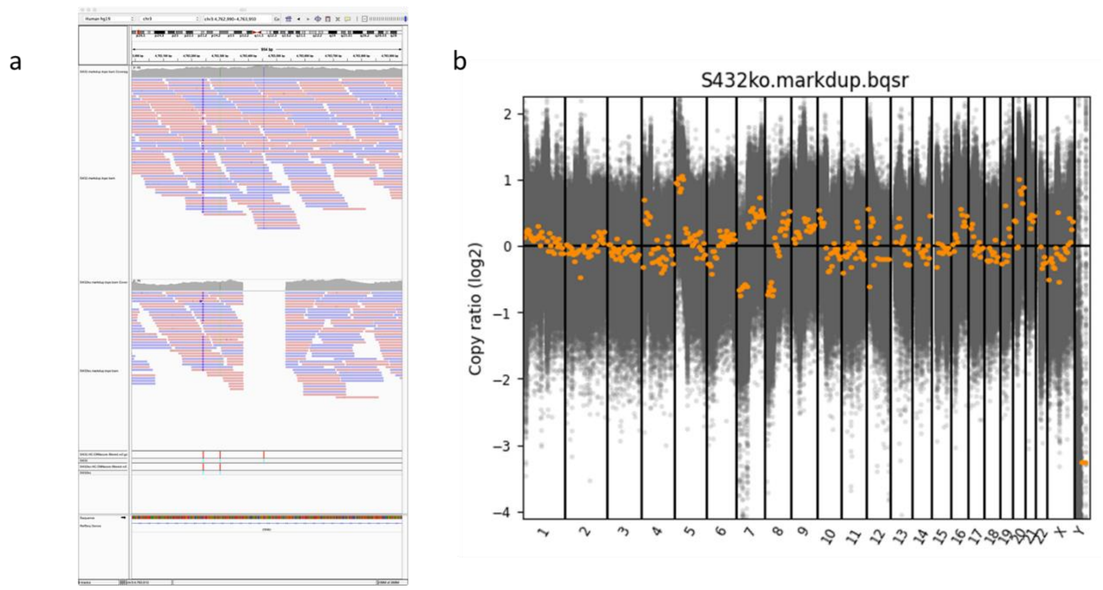

Supplementary Fig. S13. Whole genome-sequencing on the wild-type and knockout clones. Whole genome sequencing results on a wild-type (WT) and a rs9311399 knockout (KO) clones. In all 169 potential off-target sites predicted by CRISPOR, the genotypes of two samples are exactly the same. **a** Comparison of SNV and indels at KO locus indicated no sequence difference except the ~150bp KO region of rs9311399-associated enhancer. **b** No evident differences of copy number variations between WT and KO samples.

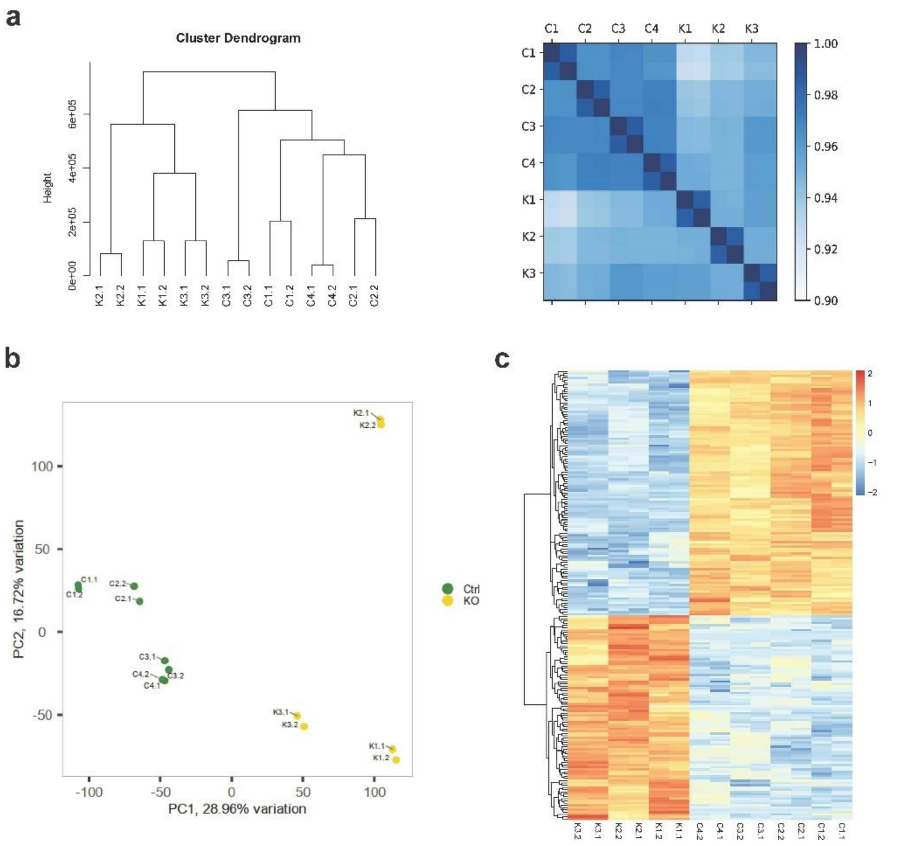

Supplementary Fig. S14. The gene expression profile of knockout cells differs from wild-type OVCA432 cells. a Hierarchical clustering analysis and heatmap of RNA-sequencing data. C1.1, C1.2, C2.1, C2.2, C3.1, C3.2, C4.1, C4.2 were wild-type (WT) OVCA432 cells. K1.1, K1.2, K2.1, K2.1, K3.1, K3.2 were knockout (KO) cells. Each clone had two technical replicates, such as K1.1 and K1.2. **b** PCA plot was based on the first two principal components of the RNA-seq results. Blue dots indicate WT cells and purple dots indicate rs9311399 KO cells. **c** Heatmap profiles of differentially expressed genes (adjusted $P < 0.05$ and $|\log2foldchange| > 1$) between WT and KO cells.

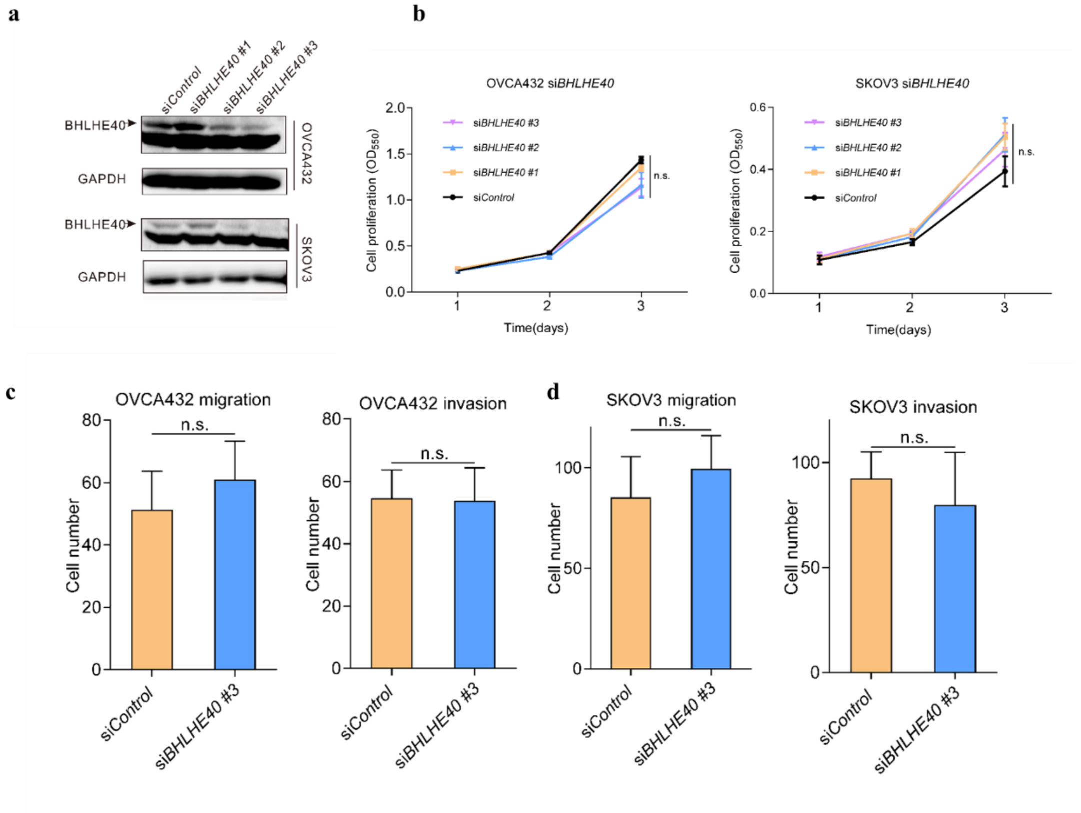

Supplementary Fig. S15. Knockdown of *BHLHE40* could not significantly affect cell growth, migration and invasion. a Knockdown of *BHLHE40* using siRNA and validated by immunoblotting with antibodies against the indicated proteins. BHLHE40 band was marked by a black arrow. **b** Cell proliferation after siRNA-mediated knockdown of *BHLHE40* expression in OVCA432 and SKOV3cells. **c** and **d** Migration and invasion assay after siRNA-mediated knockdown of *BHLHE40* expression level in OVCA432 and SKOV3 cells using si*BHLHE40* #3. Statistical comparisons of cell proliferation or cell numbers were undertaken using Student's t-tests. Data are shown as mean±s.d. n.s., not significant ($P > 0.05$).

Supplementary Table S1. Summary statistics of 31 leading SNPs with suggestive evidence for association with overall survival ($P<1\times10^{-5}$) from the discovery stage

SNP	Chr	Position [a]	G/I [b]	RA/EA [c]	SOCS			TOCS			Combined		
					EAF [d]	HR (95% CI) [e]	*P* [e]	EAF [d]	HR (95% CI) [e]	*P* [e]	EAF [d]	HR (95% CI) [e]	*P* [e]
rs376455148	2	13721886	I	A/C	0.6701	0.62(0.51-0.76)	1.41E-06	0.6724	0.91(0.73-1.13)	0.3945	0.6711	0.74(0.64-0.85)	2.90E-05
rs77849834	11	79207761	I	T/C	0.7980	0.62(0.51-0.75)	1.42E-06	0.7934	1.13(0.88-1.44)	0.3335	0.7962	0.78(0.67-0.91)	1.46E-03
rs7631664	3	4751252	G1-3	A/G	0.1525	1.60(1.32-1.94)	1.77E-06	0.1517	1.56(1.24-1.95)	1.23E-04	0.1521	1.58(1.37-1.83)	8.90E-10
rs6491919	13	105714901	I	G/A	0.3194	1.71(1.37-2.13)	2.16E-06	0.3316	0.96(0.75-1.24)	0.7768	0.3247	1.38(1.16-1.63)	1.76E-04
rs111975428	2	208657800	I	A/G	0.7559	0.62(0.51-0.76)	3.36E-06	0.7580	1.14(0.90-1.45)	0.2876	0.7568	0.80(0.68-0.93)	4.12E-03
rs147704083	1	203727055	I	A/G	0.9012	0.55(0.43-0.71)	3.45E-06	0.8954	0.97(0.69-1.36)	0.8656	0.8992	0.69(0.56-0.84)	2.87E-04
rs2582403	8	57404127	I	G/A	0.5842	1.46(1.24-1.72)	3.49E-06	0.5923	1.06(0.88-1.26)	0.5528	0.4122	1.27(1.12-1.43)	1.14E-04
rs11940381	4	133011030	I	T/G	0.6328	0.69(0.59-0.81)	4.14E-06	0.6337	0.98(0.82-1.17)	0.8146	0.6332	0.82(0.73-0.92)	9.77E-04
rs11392719	1	64258450	I	TA/T	0.2298	1.51(1.27-1.81)	4.19E-06	0.2258	0.97(0.78-1.21)	0.8167	0.2282	1.27(1.11-1.45)	6.38E-04
rs34614984	8	57401007	I	TAC/T	0.5847	1.45(1.24-1.71)	4.29E-06	0.5933	1.06(0.88-1.26)	0.5502	0.5885	1.26(1.12-1.42)	1.26E-04
rs10107586	8	142647889	G1-3	A/G	0.2876	0.65(0.54-0.78)	4.39E-06	0.2884	1.09(0.91-1.31)	0.3342	0.288	0.85(0.74-0.96)	1.05E-02
rs200717655	4	132190127	I	A/G	0.1515	0.54(0.41-0.71)	4.60E-06	0.1464	0.95(0.71-1.26)	0.7107	0.1491	0.70(0.58-0.85)	3.19E-04
rs138881643	5	158528326	I	CTTTA/C	0.5748	0.69(0.59-0.81)	4.60E-06	0.5821	0.92(0.76-1.10)	0.3601	0.5779	0.84(0.74-0.95)	4.29E-03
rs66559891	8	57404269	I	TAAACA/T	0.5873	1.46(1.24-1.71)	4.67E-06	0.5957	1.06(0.89-1.27)	0.5117	0.5910	1.27(1.12-1.43)	1.13E-04
rs76359652	5	158527393	I	A/G	0.5738	0.69(0.59-0.81)	4.69E-06	0.5818	1.12(0.93-1.35)	0.2346	0.5772	0.85(0.75-0.95)	6.94E-03
rs59064919	7	77686377	I	T/TTA	0.4095	0.64(0.53-0.78)	4.92E-06	0.4049	1.00(0.81-1.23)	0.9971	0.4074	0.78(0.68-0.90)	7.50E-04
rs4989273	2	77762221	G2	C/T	0.8664	0.58(0.46-0.73)	5.66E-06	0.8568	0.99(0.73-1.33)	0.9262	0.8627	0.72(0.60-0.86)	4.58E-04
rs2429471	12	47196461	I	C/T	0.7343	1.55(1.28-1.87)	5.88E-06	0.7414	1.03(0.84-1.28)	0.7513	0.7375	1.29(1.12-1.49)	3.38E-04
rs79270750	8	57400122	I	ATATAT/A	0.5837	1.45(1.23-1.70)	6.11E-06	0.5929	1.06(0.88-1.27)	0.5356	0.5878	1.26(1.12-1.42)	1.48E-04
rs11220499	11	126320407	I	T/C	0.3866	0.67(0.56-0.80)	7.25E-06	0.3824	0.97(0.79-1.18)	0.7565	0.3848	0.81(0.71-0.92)	1.58E-03
rs34129015	7	77665943	I	G/GT	0.1087	1.72(1.35-2.17)	7.31E-06	0.1076	0.78(0.56-1.09)	0.1444	0.1083	1.32(1.09-1.60)	4.75E-03
rs6797809	3	169672302	G2	A/G	0.8004	0.66(0.54-0.79)	7.39E-06	0.7952	0.97(0.79-1.20)	0.7836	0.7982	0.78(0.68-0.89)	3.60E-04
rs201262201	3	29668791	I	C/G	0.9394	4.18(2.23-7.83)	7.85E-06	0.9397	1.22(0.76-1.97)	0.405	0.9396	1.48(1.02-2.17)	4.13E-02
rs77141673	12	31620079	I	AG/A	0.8559	0.62(0.50-0.76)	8.02E-06	0.8544	0.99(0.77-1.28)	0.9455	0.8553	0.75(0.64-0.88)	5.48E-04
rs142897723	8	8021710	I	G/GA	0.5844	0.59(0.46-0.74)	8.15E-06	0.6128	0.77(0.58-1.00)	0.05472	0.5964	0.66(0.55-0.78)	3.54E-06
rs2428928	7	77665144	I	C/T	0.5234	1.43(1.22-1.67)	8.55E-06	0.5191	0.92(0.77-1.00)	0.376	0.5215	1.18(1.05-1.33)	5.68E-03
rs144734760	12	47182405	I	A/AAC	0.2623	0.65(0.54-0.78)	8.77E-06	0.2553	0.97(0.78-1.20)	0.7619	0.2592	0.78(0.67-0.89)	4.62E-04
rs76137490	11	126326896	I	T/C	0.7522	0.68(0.57-0.80)	9.04E-06	0.7591	1.14(0.92-1.41)	0.2344	0.7549	0.83(0.73-0.95)	6.61E-03
rs34594658	12	47189613	I	A/AT	0.7343	1.53(1.27-1.85)	9.36E-06	0.7414	1.03(0.83-1.27)	0.818	0.7375	1.28(1.11-1.47)	5.47E-04
rs178903	2	111722638	I	G/A	0.9137	0.59(0.46-0.74)	9.46E-06	0.9013	1.09(0.81-1.47)	0.558	0.0912	0.75(0.62-0.90)	2.02E-03
rs7793800	7	146362822	I	G/A	0.7012	1.53(1.27-1.85)	9.86E-06	0.699	0.99(0.82-1.22)	0.9898	0.7001	1.25 (1.09-1.44)	1.31E-03

Abbreviations: SOCS, Shanghai Ovarian Cancer Study; TOCS, Tianjin Ovarian Cancer study.

[a] hg19 position

[b] G/I: Genotyped or imputed. G1-3, genotyped with Illumina zhonghua-chip, oncoArray-chip and GSA-chip; G2, genotyped with oncoArray-chip; I, imputed.

[c] RA, reference allele; EA, effect allele.

[d] EAF, frequency of effect allele.

e Hazard ratios (HRs) and *P* values were calculated using multivariable-adjusted Cox regression under a log-additive genetic model, adjusting for the top three principal components of population stratification. CI: confidence interval

Supplementary Table S2. Associations between rs7631664 and overall survival, stratified by ovarian cancer prognostic factors

Prognostic factor	Genotypes (AA/AG/GG)	HR (95% CI) [a]	*P*	HR (95% CI) [b]	*P*	*P* for interaction [c]	*P* for heterogeneity [d]
Age							
<55	762/305/18	1.75 (1.40, 2.18)	9.28E-07	1.71 (1.36, 2.13)	3.08E-06	0.236	0.169
≥55	740/273/31	1.42 (1.17, 1.72)	3.23E-04	1.43 (1.18, 1.73)	3.13E-04		
Stage							
I-II	389/126/12	1.56 (1.03, 2.34)	0.034	1.54 (1.03, 2.30)	0.037	0.911	0.956
III-IV	1113/452/37	1.54 (1.31, 1.80)	8.45E-08	1.52 (1.30, 1.78)	1.37E-07		
Histology							
HGSOC	1084/438/38	1.52 (1.29, 1.79)	5.94E-07	1.53 (1.29, 1.80)	5.75E-07	0.319	0.303
LGSOC	55/21/0	0.76 (0.20, 2.83)	0.682	1.44 (0.35, 5.91)	0.609		
ENOC	158/54/7	1.61 (1.05, 2.47)	0.029	1.47 (0.96, 2.25)	0.079		
CCOC	58/23/2	3.73 (1.23, 10.71)	0.014	2.34 (0.78, 7.06)	0.130		
MOC	64/19/2	2.26 (1.05, 4.85)	0.038	1.86 (0.87, 3.99)	0.113		
Types							
Type 1	363/121/11	1.84 (1.33, 2.55)	2.48E-04	1.76 (1.28, 2.43)	5.41E-04	0.222	0.303
Type 2	1085/439/38	1.52 (1.29, 1.79)	5.95E-07	1.53 (1.29, 1.80)	5.76E-07		

Abbreviation: HGSOC, High-Grade Serous Ovarian Cancer; LGSOC, Low-Grade Serous Ovarian Cancer; ENOC, ENdometrioid Ovarian Cancer; CCOC, Clear Cell Ovarian Cancer; MOC, Mucinous Ovarian Cancer.

[a] Hazard ratios (HRs) were calculated within strata of each prognostic factor and were not adjusted for other prognostic factors. CI: confidence interval

[b] HR was adjusted by stage and age when appropriate

[c] Statistical significance based on a two-sided likelihood ratio test with 1 df.

[d] Heterogeneity was evaluated by Cochran's Q-test.

Supplementary Table S3. Associations between rs7631664 and overall survival from the validation study

Genotypes	N (%)	MST [a]	HR (95% CI) [b]	*P*	HR (95% CI) [c]	*P*
AA	228 (75.0)	84	Reference		Reference	
AG	73 (24.0)	69	1.72 (0.98-3.02)	0.061	1.73 (0.98-3.05)	0.058
GG	3 (1.0)	51	2.17 (0.78-5.81)	0.141	2.15 (0.79-2.90)	0.135
Additive model			1.77 (1.06-2.97)	0.029	1.79 (1.07-3.00)	0.027

[a] MST, median survival time. MST was estimated from the KM survival curve.
[b] HR, unadjusted hazard ratio. CI: confidence interval.
[c] HR was adjusted by age and clinical stage.

Supplementary Table S4. Annotation hits and fine-mapping statistics of variants in the 95% credible set.

	rs7631664	rs6781893	rs3804994	rs3804995	rs9311399
**Annotation** [a]					
E097-DNase	0	0	0	0	1
E097-H3K9me3	0	0	0	0	0
E097-H3K36me3	0	0	0	0	0
E097-H3K4me1	0	0	0	0	1
E097-H3K27ac	0	0	0	0	1
E097-H3K27me3	0	0	0	0	0
E097-H3K4me3	0	0	0	0	0
**Fine-mapping** [b]					
Zscore	-5.65166	-5.53954	-5.50512	-5.48748	-4.88212
Posterior_Prob	0.39367	0.214532	0.178484	0.162508	0.045059

[a] 1 indicates there is an annotation hit and 0 means no hits. Peak files of E097 cell line were downloaded from Roadmap (https://egg2.wustl.edu/roadmap/data/byFileType/peaks/consolidated/narrowPeak/).

[b] Annotation-based fine-mapping using PAINTOR v3.0. Zscore, the zscore of the original GWAS; Posterior_Prob, posterior probability of causality calculated by PAINTOR.

Supplementary Table S5. Significant motif scanning results based on differential binding of transcription factors in mass spectrometry

Start [a]	End [b]	Strand [c]	Variant Affinity Effect [d]	Genotype [e]	TF motif [f]	Binding *P*-value [g]	Allele Orientation [h]
4763451	4763458	+	-4.316738 -> 0.0	A/C	**HOXB4**_HM07568	A|4.82E-04	decrease binding
4763452	4763459	+	-4.096533 -> 0.0	A/C	**HOXB8**_HM07573	A|8E-04	decrease binding
4763449	4763456	-	-3.724619 -> 0.0	A/C	TOP1_HM07264	A|1.89E-03	decrease binding
4763449	4763459	+	-3.714179 -> 0.0	A/C	HOXB8_HM02317	A|1.93E-03	decrease binding
4763452	4763459	+	-3.513838 -> 0.0	A/C	TOP1_HM07265	A|3.06E-03	decrease binding
4763447	4763458	-	-3.429176 -> 0.0	A/C	HOXB4_HM05471	A|3.72E-03	decrease binding
4763438	4763457	+	-3.395786 -> 0.0	A/C	FOXA1_HM08409	A|4.02E-03	decrease binding
4763448	4763456	-	0.0 -> -3.295662	A/C	TFAP2A_HM02162	G|5.06E-03	increase binding
4763450	4763457	+	-3.200557 -> 0.0	A/C	HOXB8_HM07574	A|6.3E-03	decrease binding
4763446	4763458	+	0.0 -> -3.178792	A/C	TFAP2A_HM04044	G|6.63E-03	increase binding
4763451	4763458	+	-3.121267 -> 0.0	A/C	HNRNPA0_HM05690	A|7.56E-03	decrease binding
4763446	4763456	-	-3.04245 -> 0.0	A/C	CEBPD_HM05159	A|9.07E-03	decrease binding
4763448	4763459	-	-3.001952 -> 0.0	A/C	CEBPD_HM03140	A|9.96E-03	decrease binding

[a] Start: Motif sequence start position.
[b] End: Motif sequence end position.
[c] Strand: Strand of matched motif sequence.
[d] Variant Affinity Effect: Allele-specific effect estimated by GWAS4D with logarithm transformation *P*-value (allele1 -> allele2), the greater the negative value means stronger binding affinity.
[e] Genotype: The allele1/allele2 of rs9311399 used for motif scanning and binding affinity estimation.
[f] TF motif: The scanning TF motif with significant binding and affinity change on different alleles of rs9311399.
[g] Binding P-value: The allele and significance for allele-specific binding estimated by GWAS4D.
[h] Allele Orientation: Orientation of allele influence binding affinity.
